# Relative Effects of Dietary Administration of a Competitive Exclusion Culture and a Synbiotic Product, Age and Sampling Site on Intestinal Microbiota Maturation in Broiler Chickens

**DOI:** 10.3390/vetsci8090187

**Published:** 2021-09-06

**Authors:** Nikoletta Such, Valéria Farkas, Gábor Csitári, László Pál, Aliz Márton, László Menyhárt, Károly Dublecz

**Affiliations:** 1 Institute of Physiology and Nutrition, Department of Animal Nutrition and Nutritional Physiology, Georgikon Campus, Deák Ferenc Street 16, Hungarian University of Agriculture and Life Sciences, 8360 Keszthely, Hungary; such.nikoletta.amanda@phd.uni-mate.hu (N.S.); farkas.valeria@uni-mate.hu (V.F.); csitari.gabor@uni-mate.hu (G.C.); pal.laszlo@uni-mate.hu (L.P.); marton.aliz@uni-mate.hu (A.M.); 2 Institute of Technology, Georgikon Campus, Deák Ferenc Street 16, Hungarian University of Agriculture and Life Sciences, 8360 Keszthely, Hungary; menyhart.laszlo@uni-mate.hu

**Keywords:** gut microbiota development, ileal chymus, ileal mucosa, caecal chymus, competitive exclusion, Broilact, synbiotic, IBD antibody titre

## Abstract

In this research, the effects of early post-hatch inoculation of a competitive exclusion product (Br) and the continuous feeding of a synbiotic supplement (Sy) containing probiotic bacteria, yeast, and inulin on the production traits and composition of ileal chymus (IC), ileal mucosa (IM), and caecal chymus (CC) microbiota of broiler chickens were evaluated. The dietary treatments had no significant effects on the pattern of intestinal microbiota or production traits. The digestive tract bacteriota composition was affected mostly by the sampling place and age of birds. The dominant family of IC was Lactobacillaceae, without change with the age. The abundance of the two other major families, Enterococcaceae and Lachnospiraceae decreased with the age of birds. In the IM, Clostridiaceae was the main family in the first three weeks. Its ratio decreased later and Lactobacillaceae became the dominant family. In the CC, Ruminococcaceae and Lachnospiraceae were the main families with decreasing tendency in the age. In IC, Br treatment decreased the abundance of genus *Lactobacillus*, and both Br and Sy increased the ratio of *Enterococcus* at day 7. In all gut segments, a negative correlation was found between the IBD antibody titer levels and the ratio of genus *Leuconostoc* in the first three weeks, and a positive correlation was found in the case of *Bifidobacterium*, *Rombutsia*, and *Turicibacter* between day 21 and 40.

## 1. Introduction

The gastrointestinal tract (GIT) of poultry is colonized by a diverse group of bacterial, fungal, and protozoan species, including more than 900 bacterial species in the GIT of broilers [1]. The host maintains a synbiotic relationship with its microbial inhabitants, in which the microbes play several beneficial roles. The gut microbiota provides protection against pathogenic bacteria involved in the digestion and utilization of nutrients and helps in the normal development of gut morphology. Furthermore, metabolites and fermentation by-products of microbes are important for preserving gut health and supporting the intestinal immune maturation and homeostasis [2,3].

Complex interactions between microorganisms, ingested feed nutrients, and the GIT influence the stability and balance of microbial communities, the health of animals, growth, and, consequently, the efficiency of whole production [1,4].

In order to support the establishment and maintenance of balanced gut microbiota, lots of feed additives, such as organic acids, probiotics, or prebiotics can be used [5,6].

The competitive exclusion (CE) products composed of stable, mixed microbes derived from the intestinal microbiota of healthy adult animals and their application is based on the so-called Nurmi concept [7]. The inoculation of CE products in ovo or directly upon hatch may be a viable method to aid in the early development of a microbial population and to prevent the intestinal colonization of pathogens [8,9,10]. Based on the microbiota analysis of caecal and ileal content of chickens, the CE product Aviguard^®^ accelerated the maturation of caecal microbiota [10]. Furthermore, the product strengthened the immune system by increasing the percentages of intestinal IL-2Rα + NK cells and activating NK cells, compared to the control chickens of the same age. A similar commercial CE product inoculated in ovo enhanced the development of intestinal microbiota of broilers while decreased the abundance of undesirable bacterial species [9].

In contrast to a single inoculation of microbes at an early age of birds, the continuous feeding of pro- and/or prebiotics during the whole fattening period is another possible way to stimulate the growth and activity of beneficial microflora in the digestive tract. The most commonly used probiotics in the poultry industry contain mostly bacterial species of the Lactobacillus, Bifidobacteria, Bacillus genera or yeast of the Saccharomyces genus [11]. According to the literature, the use of multistrain probiotics is more efficient than using monostrain probiotics, because different strains of the genus show the symbiosis and additive relationships towards each other, which positively affects the microbial community [12]. Similarly, many authors agree that a synbiotic product consisting of a combination of synergistically interacting probiotics and prebiotics may provide a better efficacy in the stimulation of intestinal microbiota and protection of animal health compared to the separate application of probiotics and prebiotics [13,14,15]. Slizewska et al. (2020) evaluated the effects of three newly elaborated synbiotic preparations and two commercial probiotic products on the intestinal microbiota of broiler chickens [15]. The tested synbiotics contained strains of Lactobacillus spp., Saccharomyces cerevisiae, inulin, and had a more beneficial effect on the chickens’ health than the two probiotics alone. As with CE products, probiotics can also positively influence the responses of the intestinal immune system [16].

Much research has been done with lots of products; however, there is a lack of information on the comparison of the two previously mentioned strategies on the microbiota development within the same flock. Therefore, the aim of our study was to evaluate the effects of an early post-hatch inoculation of a CE product (Broilact^®^) and the continuous feeding of a synbiotic supplement (Bacillus subtilis, Saccharomices cerevisiae boulardii, and inulin) during the whole fattening period for the development of gut microbiota of broiler chickens. The detailed changes of microbiota composition and the diversity of ileal chymus (IC), ileal mucosa (IM), and caecal chymus (CC) were evaluated at day 7, 21, and 40. Furthermore, the relationship between intestinal microbiota and the immune response of birds to infectious bursal disease (IBD) vaccination was also investigated.

## 2. Materials and Methods

### 2.1. Animal Experiment and Dietary Treatments

A floor pen trial was carried out at the experimental farm of the Institute of Physiology and Nutrition, Hungarian University of Agriculture and Life Sciences (Georgikon Campus, Keszthely, Hungary). All husbandry and euthanasia procedures were performed in accordance with the Hungarian Government Decree 40/2013 and in full consideration of animal welfare ethics. The animal experiment was approved by the Institutional Ethics Committee (Animal Welfare Committee, Georgikon Campus, Hungarian University of Agriculture and Life Sciences) under the license number MÁB-9/2019.

A total of 432 Ross 308 one-day-old male broiler chickens were vaccinated against infectious bronchitis (CEVAC BRON), Newcastle disease (CEVAC VITAPEST) and infectious bursal disease (IBD) (CEVAC TRANSMUNE) in the hatchery (Gallus Ltd., Devecser, Hungary). In ovo IBD vaccination was used on the 19th day of incubation with automatic equipment (Embrex Inovoject, Zoetis Inc., Parsippany NJ, USA). Day-old chickens were transported to the experimental farm and randomly allocated into three treatment groups with 6 replicate floor pens of 24 chickens per pen. A maize–soybean-based basal diet was fed without feed additive in the control group (C). Birds of the second treatment (Br) were fed the control diet, and the solution of the product Broilact^®^ (Orion Corporation, Orionintie 1A, 02101 Espoo, Finland) was given to the birds via crop inoculation in two equal doses (1.25 × 10^7^ CFU/0.5 mL) at day 0 and 1. All the chickens of the two other treatment groups were inoculated with drinking water. The product Broilact^®^ (Europharmavet Ltd., H-1077 Budapest, Rózsa str. 10–12., Hungary) is a refined gut microbiota derived from healthy adult hens and was screened to ensure the absence of specific pathogens [17]. The basal diet was supplemented with a synbiotic additive mixture in the third treatment group (Sy) and fed throughout the whole trial. The synbiotic additive mixture contained three products: GalliPro^®^200, at a dose of 0.4 g/kg diet (*Bacillus subtilis*, DSM17299 bacterial strain; 1.6 × 10^6^ CFU/g, Biochem Ltd., Küstermeyerstrasse 16. 49393 Lohne, Germany); Orafti^®^ HSI containing inulin, at a dose of 5 g/kg diet (Beneo Ltd., Aandorenstraat 1, B. 3300 Tienen, Belgium); A yeast-product, Levucell^®^ SB20, at a dose of 0.05 g/kg diet, providing 1 × 10^9^ CFU viable yeast cells per kg of diet (*Saccharomyces cerevisiae boulardii*, 2 × 10^10^ CFU/g, Lallemand Ltd., Ottakringer Str. 89, A-1160 Vienna, Austria).

Three phases of fattening were used. The starter diets (0–10 days) were fed in mash; the grower (11–24 days) and finisher feeds (25–40 days) were in pelleted form. Cold pelleting was used without hydrothermal pretreatment, and the temperature of the pellets were below 60 °C. Feed and water were available ad libitum. Diets were formulated to be isoenergetic and isonitrogenous, and the nutrient content of diets met the requirements of Ross 308 broiler chickens (Aviagen, 2019). The composition and nutrient content of experimental diets is shown in Table S1. Computer-controlled housing and climatic conditions were maintained during the trial according to the breeder’s recommendations [18].

### 2.2. Measurements and Sample Collection

During the 40-day-long fattening period, the body weight (BW) of all animals was measured at day 0 and at the end of each phase. Feed intake (FI), body weight gain (BWG), and feed conversion ratio (FCR) were calculated on pen basis for each phase and for the entire trial period. On days 7, 14, 21, and 40 (d 7, d 14, d 21, d 40), 2 chickens per pen, 12 birds per treatment, were selected randomly, slaughtered, and blood and digesta samples collected. Blood samples were centrifuged at 5000× *g* for 10 min at 10 °C, and the serum was separated and stored at 5 °C until analysis. The collected serum samples were analysed for Gumboro antibody titres using an IBD specific ELISA kit (ID VET, ID Screen, IBD indirect test), which measures IgG and IgM antibodies. Ileal chymus (IC) and ileal mucosa (IM) samples were taken from a 10 cm long ileal segment, starting 3 cm after the Meckel’s diverticulum. Caecum chymus (CC) samples were collected from the left sac. Ileal and caecal contents were pushed out gently without damaging the gut structure. The luminal contents were homogenized with sterile cell spreaders and about a 2 g sample was taken into a sterile container. After the gut content collection, the ileal part was washed with sterile, ice-cold phosphate buffer solution (PBS) until the mucosa was completely cleaned from the digesta. Mucosa samples were collected aseptically by scraping off the mucosa from the internal wall of the ileal part with a glass slide. The samples were homogenized as described earlier at the ileal sampling. All samples were immediately snap-frozen in liquid nitrogen and stored at −80 °C until analysis. Before DNA extraction, the samples of two birds of the same pen were pooled. Thus, the microbiota analysis of each gut segments was carried out in 6 replicates. The actual microbial composition of Broilact was also determined in three replicates.

### 2.3. DNA Extraction, 16S rRNA Gene Amplification and Illumina MiSeq Sequencing

Bacterial DNA was extracted from 15 mg samples using the AquaGenomic Kit (MoBiTec Gmbh, Göttingen, Germany) and further purified using KAPA PureBeads (Roche. Basel. Switzerland) according to the manufacturer’s protocols. The concentration of genomic DNA was measured using a Qubit 3.0 Fluorometer with a Qubit dsDNA HS Assay Kit (Thermo Fisher Scientific Inc., Waltham, MA, USA). Bacterial DNA was amplified with tagged primers (forward 5′TCGTCGGCAGCGTCAGATGTGTATAAGAGACAGCCTACGGGNGGCWGCAG and reverse 5′GTCTCGTGGGCTCGGAGATGTGTATAAGAGACAGGACTACHVGGGTATCTAATCC) covering the V3–V4 region of the bacterial 16S rRNA gene [19]. Polymerase chain reactions (PCR) and DNA purifications were performed according to Illumina’s demonstrated protocol (Illumina Inc., San Diego, CA, USA, 2013). The PCR product libraries were quantified and qualified by using High Sensitivity D1000 ScreenTape on TapeStation 2200 instrument (Agilent Technologies, Santa Clara, CA, USA). Equimolar concentrations of libraries were pooled and sequenced on an Illumina MiSeq platform using a MiSeq Reagent Kit v3 (600 cycle; Illumina Inc., San Diego, CA, USA) 300-bp read length paired-end protocol. Raw sequence data of 16S rRNA metagenomics analysis were deposited in the National Center for Biotechnology Information (NCBI) Sequence Read Archive, under the BioProject identifier PRJNA723698.

### 2.4. Bioinformatics and Statistical Analyses

Bacteria were identified by the analysis of the V3–V4 region of the 16S rRNA gene using Illumina MiSeq platform. Sequences were analysed using Quantitative Insights Into Microbial Ecology 2 (QIIME2), version 2020.2 software package [20]. Sequences were filtered based on quality scores and the presence of ambiguous base calls using the quality-filter q-score options. Representative sequences were found using a 16S reference as a positive filter, as implemented in the deblur denoise-16S method. Sequences were clustered into Operational Taxonomic Units (OTUs) using vsearch algorithm open-reference clustering, based on a 97% similarity to the SILVA (release 132) reference database [21]. Alpha diversity metrics (Chao1, Shannon, and Simpson) and beta diversity metrics (Bray–Curtis dissimilarity) were estimated using qiime2-diversity and Calypso (San Francisco, CA, USA) online software (Version 8.84; [22]) after samples were rarefied to 1000 sequences per sample. The Chao1 was used to estimate species richness; Shannon’s and Simpson’s indexes were each used to indicate species diversity. Beta diversity analysis is used to compare the differences of sample groups in terms of species diversity. To examine differences in microbial community structure between samples, principal coordinate analysis (PCoA) with Bray–Curtis dissimilarity was generated using the Calypso online software. To verify the significance of bacterial community, an analysis of similarities calculations (ANOSIM) were performed with 999 permutations.

Statistical analysis was performed with SPSS statistical software version 23.0 (IBM Corp. Released 2015) and Calypso. The production parameters were evaluated with one-way analysis of variance (ANOVA). The differences were considered significant at a level of *p* ≤ 0.05. Data were expressed as means ± SEM. Alpha diversity indices and microbial composition at different taxonomical levels and in different intestinal samples (IC, IM and CC) were compared using two-way ANOVA test with Tukey’s HSD multiple group comparison’s post hoc test, using the dietary treatments (C, Br and Sy) and the age of birds (7, 14, 21, and 40 day) as main factors. Dietary treatments that effected each sampling time were also evaluated by one-way ANOVA. Normality of data (Shapiro–Wilk test) and homogeneity of variances (Levene’s test) were checked prior to statistical testing. Benjamini–Hochberg false discovery rate (BH-FDR) correction (FDR *p*-value) was used to adjust for multiple testing. Statistical significance was defined as FDR *p* < 0.05, whereas FDR *p*-value between 0.05 and 0.10 was considered as a trend.

Correlations between gut microbial composition and the change of IBD virus titres of individual birds were evaluated with Spearman’s correlation (Calypso, San Francisco, CA, USA). All *p*-values were calculated using two-sided tests and corrected with BH-FDR correction. An FDR *p*-value less than 0.05 was considered statistically significant.

BugBase dataset [23] was used to predict organism-level microbiota phenotypes using the OTU table from the closed OTU picking approach.

## 3. Results

### 3.1. Production Traits of Birds

Dietary treatments did not affect the body weight of broilers significantly (*p* > 0.05). Similarly, body weight gain, feed intake, and the feed conversion ratio of animals were not influenced by the dietary treatments in the starter, grower, and finisher phases, as well as for the whole trial (Table 1 and Table 2).

### 3.2. Determination the Composition of the Competitive Exclusion Product (Broilact^®^)

The Broilact^®^ (1.25 × 10^7^ CFU/0.5 mL) suspension contained mainly members of phyla Proteobacteria (43.15–44.06%), Firmicutes (43.33–43.79%), Bacteroidetes (11.94–13.19%), and Actinobacteria (0.21–0.33%). At genus level, the main bacteria groups were *Escherichia-Shigella* (42.2–43.14%), *Enterococcus* (14.06–17.18), *Bacteroides* (11.04–12.57%), and *Lactobacillus* (6.6–8.62%) (Figure S1).

### 3.3. Alpha and Beta Diversity

**Figure 1:**
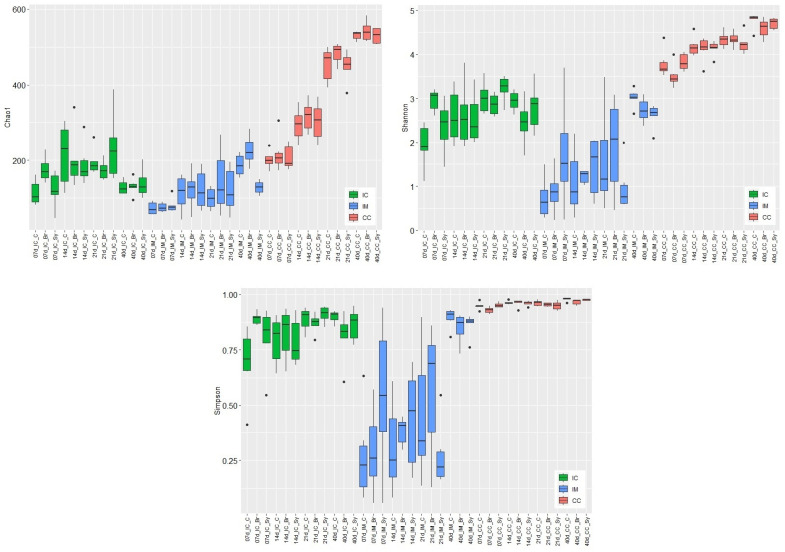
Effects of dietary treatments (C—Control, Br—Broilact^®^, Sy—Synbiotic feed additive) and age of birds on the alpha diversity of ileal chymus (IC), ileal mucosa (IM), and caecal chymus (CC) microbiota in broiler chickens. Boxplots representing alpha diversity by Chao1 estimator, Shannon, and Simpson indexes. Boxes represent the median, the 25th, and the 75th percentiles of the groups. Alpha diversity indices at different taxonomical levels and in different intestinal samples (IC, IM, and CC) were compared using two-way ANOVA test with Tukey’s HSD multiple group comparison’s post hoc test, using the dietary treatments (C, Br and Sy) and the age of birds (7, 14, 21, and 40 day) as main factors. The differences were considered significant at a level of *p* ≤ 0.05. Dots represent the outlier values.

In this study, from all 216 samples, a total of 6,242,578 good-quality 16S rRNA reads were available for analysis after quality filtering. The average sequence numbers were 27,880 in IC (min: 15,599; max: 45,828); 28,821 in IM (min: 5348; max: 61,484); 30,002 in CC (min: 9803; max: 49,534), respectively. These sequences were assigned to 1714 OTUs at 97% similarity using the open approach. Rarefaction curves for the observed OTUs approached a plateau, indicating that the sequencing depth was sufficient for the coverage of all OTUs presented in the samples (Figure S2). The rarefaction curves for species richness and Chao1, Shannon, and Simpson diversity indices demonstrated that the greatest diversity of the microbiota was present in the CC, followed by IC, and IM (Figure 1). The diversity indices were mostly influenced by the age of chickens and the sampling place, and to a lesser extent by the dietary treatments.

The increase in microbial diversity in IC reached its maximum at the end of the second (Chao1) or third week (Shannon, Simpson; Table S2). After that, the diversity decreased significantly (Chao1) or trend-like (Shannon, Simpson) until d 40. The average values of diversity indices were 132 for Chao1, 2.74 for Shannon, and 0.86 for Simpson index at d 40. The values in IC at d 40 did not differ significantly from those of d 7. The diversity of the IC microbial community was lower than that of the caecum. Evaluating the results of IC at d 7, the diversities of Br dietary treatment were significantly higher compared to the control treatment.

Microbial diversities in the IM increased continuously with the age of birds without reaching a plateau. The increase was only significant at d 40 (Table S3). Compared to the other two sampling places, according to the Simpson index, variation of diversity was the highest in the IM. Of the three intestinal sections, the microbial diversity of IM was the lowest before d 40. At d 40, Shannon and Simpson indices were higher in the mucosa, and the Chao1 index was considerably higher than those of the chymus. The average values of diversity indices were 180 for Chao1, 2.79 for Shannon, and 0.87 for Simpson index at d 40. The Chao1 index showed significant dietary treatment effect only in the IM. This index was significantly higher in the C and Br treatment groups than in the Sy group. The Shannon index showed also a significant difference between the treatments of C and Sy at d 40. According to the Shannon and Simpson indices, the interaction between dietary treatments and age were significant in IM.

In CC, the diversity of microbiota also increased continuously with the age of chickens, and significant differences were found between the different age categories (Table S4). The Simpson index showed that significant treatment was only effective in CC. In the case of the treatment in Br, the Simpson diversity was lower than that of the control group. The average values of diversity indices at d 40 were 534 for Chao1, 4.69 for Shannon, and 0.97 for Simpson index.

**Figure 2:**
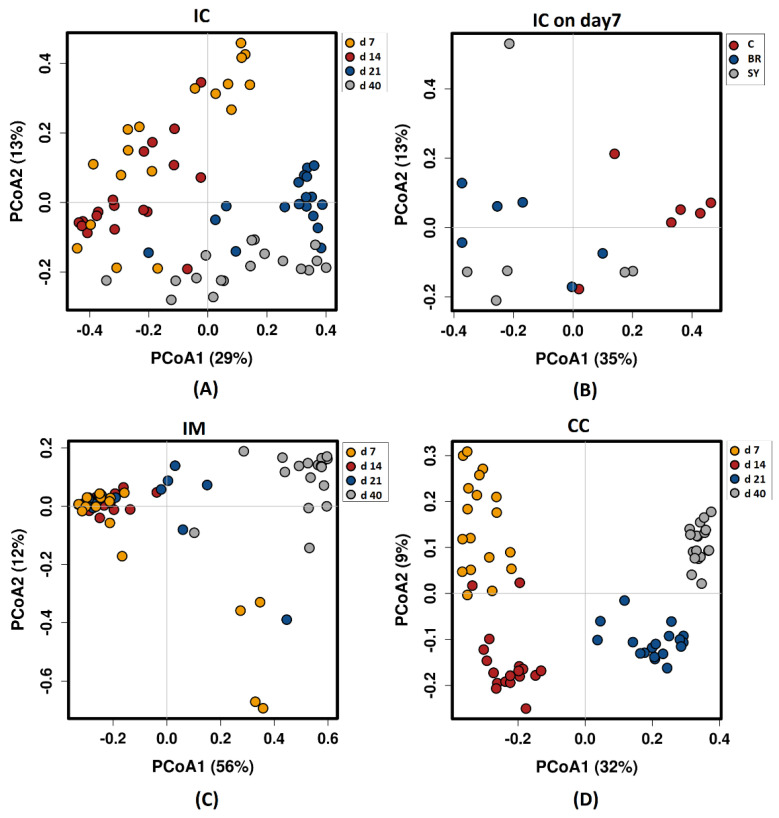
Principal coordinate analysis (PCoA) based on Bray—Curtis dissimilarity matrix on sampling sites: (**A**) Ileal chymus age effect; (**B**) Ileal chymus dietary treatment effect at d 7; (**C**) Ileal mucosa age effect; (**D**) Caecal chymus age effect. Dietary treatments were C—Control, Br—Broilact^®^, Sy—Synbiotic feed additive. The percentage of variation explained by each PCoA is indicated on the axes with Bray—Curtis dissimilarity. To verify the significance of bacterial community analysis of similarities (ANOSIM) calculations were performed with 999 permutations. The differences were considered significant at a level of *p* ≤ 0.05.

Beta-diversity based on principal coordinate analysis (PCoA) ordination using Bray–Curtis dissimilarity matrix showed a significantly different (ANOSIM global R = 0.72, *p* = 0.001) bacterial community structure among sampling places (Figure 2). The ANOSIM tests revealed statistically significant differences caused by the age of birds in all sample types. In IC (R = 0.518, *p* = 0.001), the results showed an overlap between the 7, 14, 21, and 40-day-old groups (Figure 2A). The structure of the bacterial community was not affected by the dietary treatments, except at d 7 in IC (R = 0.307, *p* = 0.002) (Figure 2B). In IM (R = 0.356, *p* = 0.001), a similar microbial community with a high overlap was found at d 7, 14, and 21; however, the bacterial community at d 40 was different (Figure 2C). The High R value in CC (R = 0.816, *p* = 0.001) suggested a high dissimilarity between the age-related bacterial structures (Figure 2D).

### 3.4. Taxonomic Composition at Phylum Level and Age-Related Changes in Gut Microbiota

**Figure 3:**
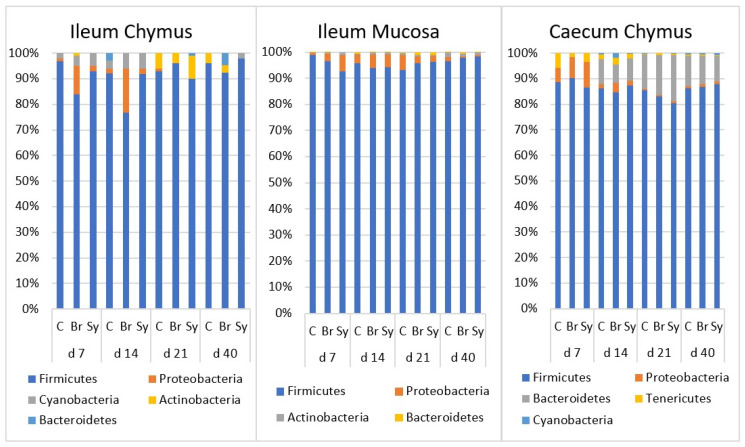
The relative abundance of microbiota at phylum level in the ileal chymus (IC), ileal mucosa (IM), and caecal chymus (CC) as affected by the age of chickens and dietary treatments (C—Control, Br—Broilact^®^, Sy—Synbiotic feed additive). Microbial composition at different taxonomical levels and in different intestinal samples (IC, IM and CC) were compared using two-way ANOVA test with Tukey’s HSD multiple group comparison’s post hoc test, using the dietary treatments (C, Br and Sy) and the age of birds (7, 14, 21, and 40 day) as main factors. The differences were considered significant at a level of *p* ≤ 0.05.

During the evaluation, the relative abundance of major (above 15%) and minor taxa (between 3 and 15%) was described. At the phylum level, both age and dietary treatments had a significant effect on the composition of gut microbiota (Tables S5–S7). The composition of microbiota at different time points are shown in taxa bar plots (Figure 3). At each time point, Firmicutes was the major dominant phylum (80.61–97.23%) in all three intestinal areas. Its relative abundance was the highest in IM. No age-related trend was found in the different sampling places.

Proteobacteria was one of the minor phyla in all three gut areas with a decreasing relative abundance over time. In IC and IM, its relative abundance was significantly higher at d 14 (*p* < 0.05) and dropped below 1% later on in IC. Its decrease was slower in the IM and decreased below 1% only at d 40. In CC, the relative abundance was significantly higher at d 7 (*p* < 0.0001) and decreased over time.

Bacteroidetes was barely detectable at d 7; its relative abundance increased continuously in the caecum until d 21 (*p* < 0.0001) and remained at 11% until d 40.

### 3.5. Taxonomic Composition at Family Level and Age-Related Changes in Gut Microbiota

**Figure 4:**
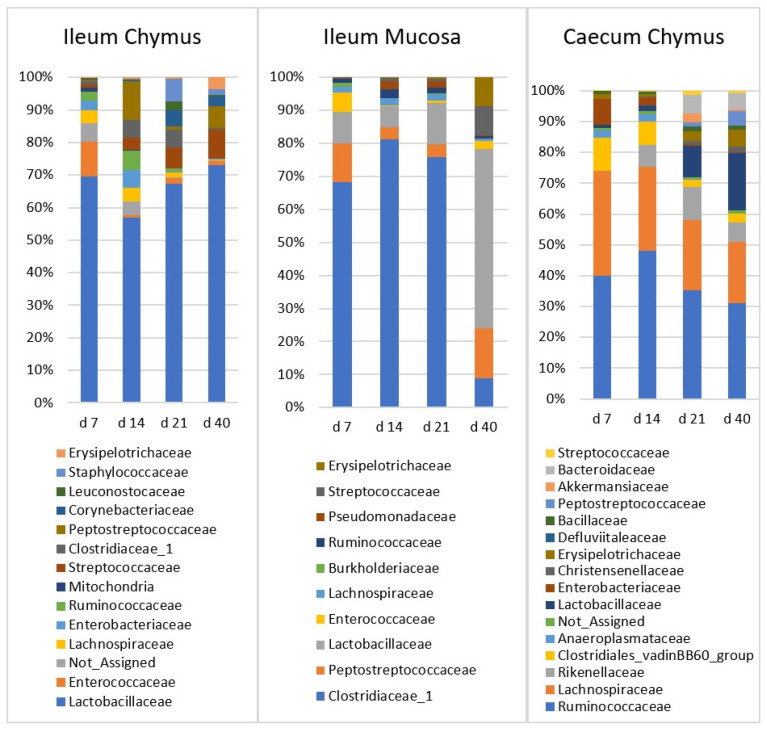
The relative abundance of microbiota at family level in the ileal chymus (IC), ileal mucosa (IM), and caecal chymus (CC) as affected by the age of chickens. Microbial composition at different taxonomical levels and in different intestinal samples (IC, IM, and CC) were compared using two-way ANOVA test with Tukey’s HSD multiple group comparison’s post hoc test, using the dietary treatments (C, Br, and Sy) and the age of birds (7, 14, 21, and 40 day) as main factors. The differences were considered significant at a level of *p* ≤ 0.05.

The detailed age and dietary treatment effects are shown in Table 3, Table 4 and Table 5. The composition of the microbiota at different time points is also shown in taxa bar plots (Figure 4).

In the IC, there was one major family (Lactobacillaceae) and ten minor families. Relative abundance of Lactobacillaceae did not change significantly with the age of chickens. Streptococcaceae and Erysipelotrichaceae families were characterized with low abundance at d 7 (1.04%, 0.10%, respectively), then their ratio increased significantly (*p* < 0.0001) until d 40 (9.17%, 3.93%, respectively). Staphylococcaceae, Corynebacteriaceae, and Clostridiaceae_1 were also present at low abundance at d 7, and reached their maximum at d 21 (7.2%, 5.73%, 6.16% respectively). The increase in Staphylococcaceae and Corynebacteriaceae was significant (*p* < 0.0001). Relative abundances of Enterococcaceae and Lachnospiraceae were the highest in the first two weeks, and both families showed decreasing trend later. The ratio of Enterobacteriacea also decreased rapidly after the first two weeks. Bacillaceae was the only family which did not show a significant age effect.

In the IM, there were three major families (Clostridiaceae_1, Peptostreptococcaceae, and Lactobacillaceae) and three minor families. Clostridiaceae_1 had the highest initial ratio (66.1%), and did not change significantly between d 7 and 21. However, at d 40, its abundance declined abruptly to 6.92% (*p* < 0.0001). In contrast, Peptostreptococcaceae and Lactobacillaceae showed an increasing relative abundance. The ratio of both families was lowest at d 14, and then increased significantly (15.6% and 50.9%; *p* < 0.022 and *p* < 0.0001, respectively) until d 40. Streptococcaceae and Erysipelotrichaceae had a similar increasing trend. The abundance of both was low at d 7 (0.19% and 0.06%) then reached the plateau at d 40 (8.96% and 8.76%, respectively). Changes of Enterococcaceae with age was not significant. Enterococcaceae declined between d 7 and 14 (from 7.42 to 0.30%) and showed no age effect afterwards.

In the CC, there were three major (Ruminococcaceae, Lachnospiraceae, and Lactobacillaceae) and seven minor families. Ruminococcaceae, from d 14, and Lachnospiraceae, the Clostridiales vadinBB60 group, Enterobacteriaceae, and Anaeroplasmataceae, from d 7, showed a decreasing trend, and the initially high frequency decreased significantly until d 40. Lactobacillaceae and two minor families, Erysipelotrichaceae and Peptostreptococcaceae, showed an inverse trend compared to the previous ones. Relative abundances of these families were significantly low at d 7, then these values continuously increased until d 40. Rikenellaceae and Bacteroidaceae showed a similar pattern, but the increase was significant only until d 21.

### 3.6. Dietary Treatment-Related Changes in Gut Microbiota Composition

**Figure 5:**
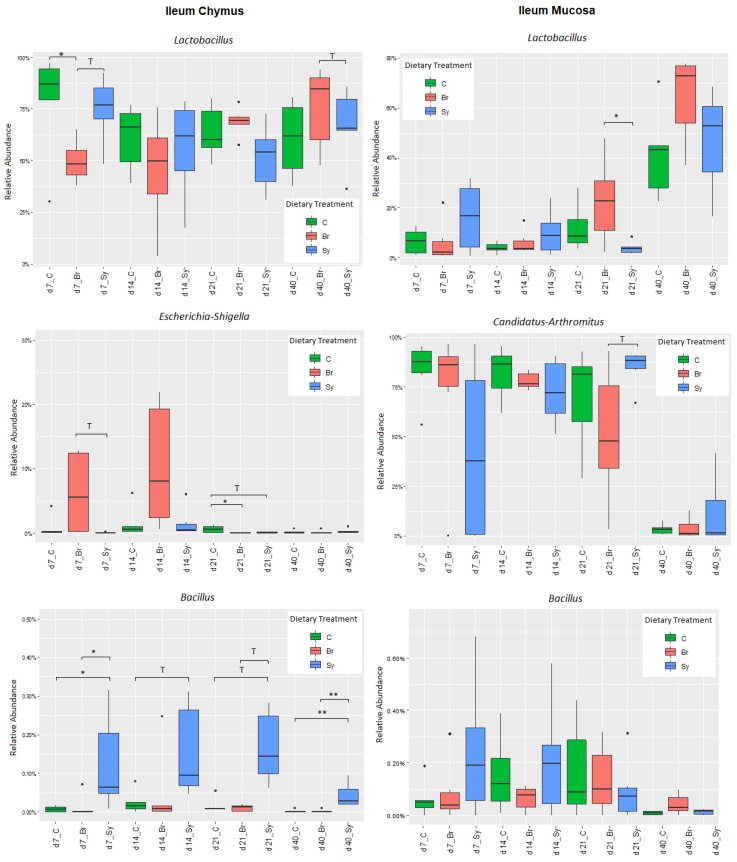
Boxplots showing the abundances of the taxonomic groups at genus level in the ileal chymus (IC) and ileal mucosa (IM) as affected by the dietary treatments and age of birds. Statistical significance was determined by one-way ANOVA and the Tukey multiple-comparisons test. Results with an adjusted *p*-value below 0.05 (* *p* < 0.05; ** *p* < 0.01; were considered statistically significant and results between 0.05 and 0.1 (0.05 < *p* < 0.10) were considered a trend (T). Dots represent the outlier values.

In the IC, the relative abundance of phyla Firmicutes at d 7 was significantly lower in the Br treatment group compared with the control. The same treatment related significant differences, which were found for Proteobacteria at d 21. On the other hand, treatment Sy resulted in a significantly higher abundance of Bacteroidetes (at d 7) and Cyanobacteria (at d 7 and 21) than the control (Table S5). In order to analyse the dietary treatment-related changes in gut microbiota in more detail, the genus level was also considered (Table S8, Figure 5). Relative abundance of genus Lactobacillus at d 7 was significantly lower, in contrast Enterococcus abundance, which was significantly higher in the Br treatment group compared with the control. Br treatment resulted a higher abundance of Escherichia-Shigella at d 7 and d 14, and its ratio decreased thereafter. The relative abundance of Bacillus was low, but treatment Sy led to a clear increase in this genus at all time intervals. The differences compared with the other treatments were significant at d 7 and d 40.

In the IM, phyla Firmicutes, Proteobacteria, Actinobacteria, and Cyanobacteria were affected by the dietary treatments at d 7 (Table S6). Sy treatment decreased the abundance of Firmicutes and increased the abundance of the other three phyla compared with the control treatment. Regarding the different genera, the only significant dietary treatment effect on Lactobacillus was found at d 21, when Br increased its abundance compared with treatment Sy (Table S9). The proportion of major genus Candidatus Arthromitus changed in an interesting way. In the first three weeks, it was present with a high abundance, but decreased sharply at d 40. Although the differences were not significant, the relative abundance of Bacillus was increased by Sy at d 7 and d 14 (Figure 5).

In the CC, no significant dietary treatment effect was found at the phylum or genus level (Tables S7 and S10). The only significant effect was the decreased relative abundance of the family Erysiphelotrichaceae at d 14 and d 40 in the Br treatment group compared with the control.

### 3.7. Bugbase

**Figure 6:**
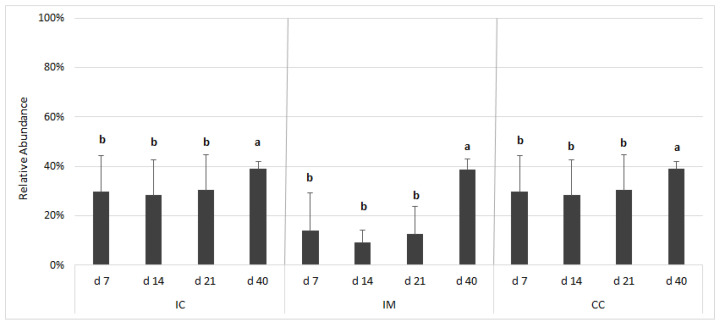
Relative abundance of potentially pathogenic bacteria according to BugBase analysis based on the 16S rRNA gene sequencing dataset. The outcome was grouped according to the gut segments and age. Gut segments were ileal chymus (IC), ileal mucosa (IM), and caecum chymus (CC). The relative abundance is presented on the y-axis, which is calculated by closed/open OTU picking ratio. Differences between groups were assessed using Mann–Whitney–Wilcoxon test, with Benjamini–Hochberg false discovery rate (FDR) correction. FDR-corrected *p*-values below 0.05 were considered significant. a, b: Averages with different letter marks differ significantly (FDR *p* < 0.05).

The closed OTU picking approach yielded a lower number of OTUs (713 vs. 1714 from the open approach) because this approach relies on an exact match against reference sequences. The relative representation of potentially pathogenic microorganisms was predicted on the basis of Bugbase database (Figure 6). The relative abundances of potential pathogens in all three sampling sites significantly differed (*p* < 0.0001) from each other. The proportion of potential pathogens was high in the IC and CC and low in the IM. There was no change until d 21, but thereafter there was a significant increase at all three sampling sites. The largest increase in the proportion of potential pathogens was observed in the IM between d 21 and d 40.

### 3.8. Infectious Bursal Disease Antibody Titres

**Figure 7:**
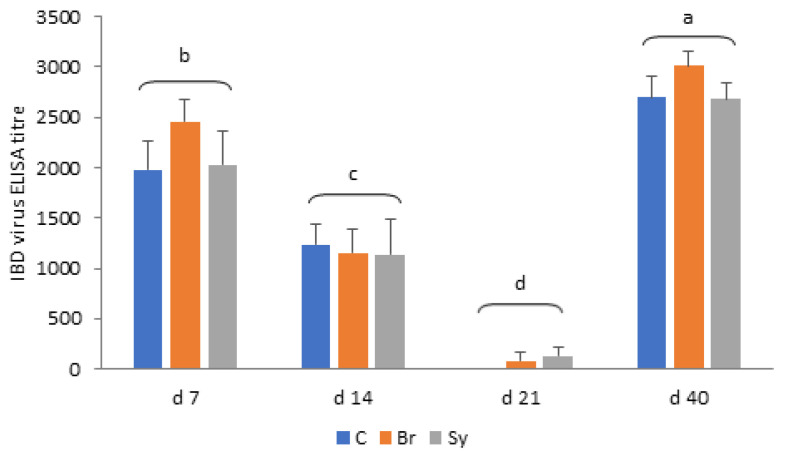
Infectious bursal disease (IBD) virus ELISA antibody titres in different treatment groups (mean ± SD). C—Control, Br—Broilact^®^, Sy—Synbiotic feed additive; All *p*-values were calculated using two-sided tests and corrected with BH-FDR correction. FDR *p*-value less than 0.05 was considered statistically significant. a, b, c, d: Averages with different letter marks differ significantly (*p* < 0.05).

As shown in Figure 7, mean IBD antibody titre values decreased from d 7 to 14 significantly, and a further significant decline was observed at d 21 in each treatment group (*p* < 0.05). The adaptive immune response of chickens increased the titre values significantly from d 21 to d 40, irrespective of treatments. There were no significant differences between the titre values of different dietary treatments at the same sampling time (*p* > 0.05).

### 3.9. Correlation between the IBD Antibody Titre Values and Microbiota

Spearman’s correlations between gut microbiota composition and IBD virus titre values of birds are shown in Supplementary Tables S11 and S12. The maternal antibody titre levels were positively correlated with the *Leuconostoc* genus in all gut segments during the first 21 d of life. In the IM, negative correlation was found with *Lysinibacillus*, and positively correlation was found with *Cutibacterium* and *Anaerobacillus*.

After the first 21 d of life, when the response of an adaptive immune system was detectable, there were strong positive correlations between IBD titres and the genera *Bifidobacterium, Rombutsia*, and *Turicibacter* in all gut segments. In addition, in the IM IBD titre values were strongly and positively correlated with the *Gallicola*, *Lactobacillus*, *Neoscardovia*, *Rothia*, and the *Ruminococcaceae UCG-008*; however, they were negatively correlated with *Candidatus Arthromitus*, *Carnobacterium*, *Delftia*, *Lysinibacillus*, *Ochrobactrum*, *Pseudomonas*, *Serratia*, and the *Stenotrophomona*.

## 4. Discussion

Different gastrointestinal tract regions of chickens play different roles in digestion, nutrient absorption, and intestinal health [24]. In the hatchery, the newly hatched chicken acquires its initial microbiota from an artificial environment instead of the natural maternal source, and this colonization is dependent upon the presence of environmental bacteria [25,26]. There is consensus in the scientific community that early colonization of the intestine is of great importance for poultry health and productivity, since it can alter the morphology and physiology of the intestine and its susceptibility to infectious diseases [27]. Therefore, in this study, we aimed to induce an alteration in the intestinal microbiota of broiler chickens by administration of a CE product and through the continuous feeding of a synbiotic feed additive.

### 4.1. Performance Parameters

In our study, none of the dietary treatments a showed significant effect on the production traits of broilers (body weight, feed intake, and feed conversion ratio). The positive results of feed additives with microbiota-stimulating actions on animal performance have not always been demonstrated, which could be explained by the various experimental conditions and pathogen challenges across the experiments. Similar to our results, the use of the Broilact treatment did not affect the body weight or growth rate of broilers in some experiments [28,29]. However, according to Schneitz (2005), several studies have been shown that Broilact treatment enhances the growth and decreases the mortality of birds and improves the feed conversion ratio [30]. The *Bacillus subtilis* DSM17299 bacterial strain used in our experiment was described by Reis et al. (2017) as improving performance and reducing production costs [31]. As was demonstrated in our experiment as well, probiotic (*Bacillus licheniformis* and *Bacillus subtilis* [32]) or synbiotic (inactivated and live *Saccharomyces cerevisiae* [33]) treatments do not always improve the performance parameters of broilers significantly. In a recent study, *Bacillus subtilis* was used in ovo, either in the feed or in the drinking water. Despite this, the in ovo treatment increased the jejunum villus height by 23%, and the treatment had no significant effect on the growth performance of broilers [34]. Increasing the absorption surface of the small intestine did not necessarily improve nutrient digestibility and growth rate, if the basic surface area was enough for the maximal absorption. However, chickens with more commensal and less potential pathogenic bacteria means that there is less chance for intestinal disorders. This improved gut health also has economic importance. The dose of the pro- and prebiotics is also crucial and it could partly explain the controversial results. As it has been shown by Kanwal et al. (2020), feeding Saccharomyces cerevisiae with broilers results in an improved growth rate only at 1.5 g/kg of the inclusion rate [35].

### 4.2. Diversity of Gut Microbiota

The diversity and composition of the GIT microbiota are influenced by many factors, including age, diet, environmental parameters, management, and feed additives. Alpha and beta diversity analyses revealed that the microbiota composition was influenced primarily by age and intestinal section. Alpha diversity values increased steadily in IM and CC with the age of chickens, which is consistent with the observations of other studies [36,37,38]. The diversity of IC microbiota showed a different pattern because it reached its maximum at d 21 and declined afterwards.

We found that the dietary treatments differently affected the richness and evenness of ileal and cecal microbial communities in broilers. In the caecum, only the Simpson index showed a significant dietary treatment effect, namely when Br treatment decreased the values of this index compared with the control. The Simpson index is less sensitive to rare species [39,40] than the Shannon index, showing that the reason for the change in diversity is not a change in the number of rare species. Furthermore, our results showed that Br treatment significantly increased the alpha diversity of IC microbiota during the early period (d 7). This changes in the microbiota measured at d 7 is also supported by the changes of beta diversity. Contrary to this, the Sy treatment slightly decreased the alpha diversity in IM later at d 40. The reason for the different dietary effects could be that Broilact was applied only immediately after hatch, while the synbiotic product was fed during the whole fattening period.

### 4.3. Effects of Age and Dietary Treatments on Composition of Intestinal Microbiota

#### 4.3.1. Ileal Chymus (IC) Microbiota

Firmicutes dominates in the microbiota of ileum [41,42,43], however the degree of dominance differs between the experiments, even between the chickens in the same treatment group [41]. Firmicutes dominance is a consequence of the dominance of lactic acid bacteria, mainly genera *Lactobacillus*, *Enterococcus*, and *Streptococcus*. Accordingly, Lactobacillaceae was the dominant family in IC throughout the development of the chickens.

The abundance of Lactobacillaceae and Enterococcaceae at d 7 were 68.2% and 11.2%, respectively. After a small decrease at d 14, Lactobacillaceae remained the most abundant family until d 40. Enterococcaceae almost disappeared by d 14 (0.72%) and did not increase significantly later. Streptococcaceae constantly increased from 1.04% at d 7 to a final 9.2% at d 40. Most studies observing bacterial composition in the ileum demonstrated different results. Early microbiomes are the most variable ones among studies [44]. Examining the changes in microbiota over chicken aging in different intestinal sections, Glendinning et al. (2019) found that genus *Lactobacillus* (50%) and *Enterococcus* (41%) were dominant in the ileum at d 7 [37]. Later, the abundance of *Enterococcus* decreased, and the dominance of lactobacilli increased. Ranjitkar et al. (2016) published similar results, wherein the major families were Lactobacillaceae (61.3%) and Enterococcaceae (25.3%) at d 8, while the typical minor families were Lachnospiraceae (6.1%) and Clostridiaceae (4.7%) in the ileum [43]. By d 36, Lactobacillaceae remained dominant (67.7%), while the second major family became Clostridiaceae (19.3%). Streptococcaceae became more abundant (4.7%) and Enterobacteriaceae slightly decreased from 0.2% to d 36 [37]. In our study, the proportion of Clostridiaceae was lower than in other publications.

In the phylum and family levels, there were significant differences at d 7 because of dietary treatments. Broilact significantly decreased the relative abundance of phylum Firmicutes and family Lactobacillaceae at d 7. The relative abundance of Enterococcaceae at d 7 in both Br and Sy dietary treatments was higher than the control, but the reason for this is not clear, as only the Broilact product contained a large amount of *Enterococcus* spp. (14–17%), and this genus is not part of the synbiotic product. After d 7, the proportions of Enterococcaceae declined rapidly in all three dietary treatments. This decrease with the age was consistent with the observations of other authors [37,43]. The proportion of *Bacillus* spp. was very low in all dietary treatments and in all sampling times in IC. However, there was a clear trend from d 7 to 21, and a significant difference at d 40, when the relative abundance of *Bacillus* spp. in the Sy treatment was higher than that in the two other dietary treatments. This means that the Sy product was able to modify the microbial composition of IC for longer time intervals.

The ratio of Proteobacteria was at least five times higher in the Br group than in the other dietary treatments at d 7. This might be explained by the high proportion (43–44%) of members of this phylum in the Broilact product. This change is not necessarily positive, because it has been described in several studies that the higher abundance of Proteobacteria in the small intestine is associated with compromised chicken performance [45]. At d 21, the relative abundance of Enterobacteriaceae in the control animals was significantly higher than that in Br birds. This difference can also be seen at the genus level, as the main member of the Enterobacteriaceae was the genus *Escherichia-Shigella*. Lactobacilli produce acetic and lactic acid, and this contributes to the inhibition of many acid-sensitive bacteria, such as members of Enterobacteriaceae, by lowering the pH of the intestinal contents [46]. During the growth of the chicken, it takes about 2 weeks for lactobacilli to become the predominant bacteria [47]. This may explain the decrease in the relative abundance of *Escherichia-Shigella*. The incidence of species of Enterobacteriaceae within the intestine is a standard marker of dysbiosis [48], even though it is well known that this family is a pioneer colonizer of the gut [26].

#### 4.3.2. Ileal Mucus (IM) Microbiota

Firmicutes was the major dominant phylum (93.7–97.8%) in IM. Among the other phyla, only the relative abundance of Actinobacteria exceeded 1% at d 40 only. This Firmicutes dominance is higher than that which is generally described in other studies [49,50]. Of the families belonging to phylum Firmicutes, Lactobacillaceae occurred at a higher frequency and Ruminococcaceae occurred at a lower frequency than it could be found in the literature [42,44].

Clostridiaceae_1 was the dominant family at early age and its relative abundance continuously decreased from 66.1% (d 7) to 6.92% (d 40). At d 40, after a continuous increase, Lactobacillaceae became the dominant family (9.58% d 7; 50.9% d40). At the genus level in our study the *Candidatus Arthromitus* was the dominant genus in IM at d 7, 14, and 21, with a significantly higher abundance in all sampling times, compared with the other sampling areas. Richards-Rios et al. (2020) also found a high abundance of *C. Arthromitus*, persisting between d 7 and 14, after which *Lactobacillus* became the most abundant genus in both the mucus and lumen [44]. In their experiment, the dominance of the ileal microbiota by *Lactobacillus* was a transient feature. At d 42, the relative abundance of *Lactobacillus* was lower, while a range of other taxa, including *Escherichia*, *Turicibacter*, and members of Clostridiales, were higher [44]. In our study, this decreasing trend of *Lactobacillus* was not observed.

Dietary treatments resulted in a significant change at the phylum level at d 7. The relative abundance of Firmicutes was the highest in treatment C and those of Proteobacteria and Actinobacteria in treatment Sy. At the family level, the only significant difference presented was Lactobacillaceae, of which the relative abundance at d 21 was lower in treatment Sy compared with treatment Br. At genus level, the relative abundance of *Lactobacillus* and *Ruminococcaceae UCG-008* were the highest in treatment Br at d 21 and 40, respectively. The relative abundance of the *Lactobacillus* genus multiplied by d 40 compared to d 7, regardless of dietary treatments. In the study of Knarreborg et al. (2008), *Bacillus subtilis* modulated the intestinal microbiota and favoured the growth of lactic acid bacteria in the ileum [51]. This phenomenon was observed only at d 7 and 14, similar to our study. In a previous study of Wang et al. (2016), when a prebiotic, a probiotic, and a synbiotic treatment was used, the prebiotic increased the ratio of *Lactobacillus* in the ileal mucosa at an early age, but the probiotic *B. subtilis* did not affect *Lactobacillus* spp. or *Escherichia coli* levels [52]. The reason for the increase in lactobacilli at d 21 in the Broilact-treated group is difficult to explain.

In our study, *Candidatus Arthromitus* was the major genus of IM in the first 3 weeks. The Sy treatment tended to decrease its relative abundance at d 7 and increased the proportion of *Lactobacillus* and *Enterococcus* genera. The explanation for these trends can be that, according to He (2019), *B. subtilis* is an aerobic bacterium that uses oxygen in the intestine to provide anaerobic environment for the colonization of anaerobic bacteria [53]. Lower oxygen levels may have caused a decrease in *C. Arthromitus* and an increase in the genus *Lactobacillus* and *Enterococcus*. Yeasts in the synbiotic product could also influence the microbial composition in the mucosa. *Saccharomyces boulardii* can adhere to the intestinal mucus, and this adhesion contributes to reducing the availability of binding sites for pathogens [54]. *C. Arthromitus* forms filaments that are anchored to ileal gut epithelial cells at the attachment sites [55,56]. The occupying attachment sites by yeast may be another reason for the lower abundance of *C. Arthromitus* in the Sy group in the first week.

#### 4.3.3. Caecum Chymus (CC) Microbiota

Generally, the microbes in the caecum belong to two major phyla, Firmicutes and Bacteroidetes, followed by two minor phyla, Actinobacteria and Protebacteria [41,57]. In our study, the caecal microbial composition showed high Firmicutes dominance (87%) with a low level of Bacteroidetes (9.9%). The Firmicutes/Bacteroidetes ratio is important for nutrient utilization. However, the metabolic way with which Firmicutes and a higher ratio of Firmicutes/Bacteroidetes in caecal microbiota improves the utilization of dietary energy is not fully understood yet [58].

In our case, Ruminococcaceae, Lachnospiraceae, and Lactobacillaceae were the main families in the caecum, constituting about 70% of the total families. Compared to the other studies [42,59,60], in our experiments, Lactobacillaceae was present at a higher percentage.

The high abundance of Ruminococceae (41.0%) and Lachnospiraceae (31.6%) at d 7 decreased constantly until d 40 (29.7% and 18.7%, respectively). Community analysis of caecal samples across time points showed that Gram-negative bacteria (Proteobacteria) dominated at very early time points (before d 7), while Gram-positive Firmicutes, especially families of Clostridia taxa (Ruminococcaceae and Lachnospiraceae), became more prominent with age [61]. Ranjitkar et al. (2016), examining the change in the composition of the caecum microbial community over chicken aging, found that the dominant families were Lachnospiraceae (39.1%), Ruminococcaceae (29.5%), and Lactobacillaceae (17.5%) in the caeca at d 8 [43]. Lachnospiraceae and Lactobacillaceae constantly decreased (22.7% and 3.3%, respectively), while Rumnococacceae increased to 36.1% until d 36. Enterobacteriaceae suddenly declined from 6.2% (d 8) to 0.5% (d 15) and continued to decline from 0.2% to d 36. Rikenellaceae increased to 26.3% at d 21 and retained its high abundance (20.2%) until d 36. Erysipelotrichaceae and Clostridiacea increased to 3.2% and 4.8%, respectively, by d 36 [43].

The dietary treatments did not cause significant change at any (phylum, family, or genus) taxonomic level in CC, with one exception. The relative abundance of Erysiphelotrichaceae at d 14 and 40 was significantly higher in the treatment group C than in treatment Br. In the study of Meijerink (2020), the inoculation of CE product immediately after hatch successfully altered intestinal microbiota composition, especially in the first week of life, but did not permanently influence the diversity of caecal microbiota [10]. According to Ma et al. (2018), increased Firmicutes and reduced Bacteroidetes abundance were observed in the caecum in response to *B. subtilis* supplementation [62]. Rodrigues and co-workers (2020) compared the effects of single probiotics, yeast, and a synbiotic treatment on the caecal microbiota modulation, and only the synbiotic treatment influenced the caecal microbial community structure at d 42 [63].

### 4.4. Bugbase

Bugbase is an algorithm that predicts the organism-level coverage of biologically interpretable phenotypes. We have found only one study [64] which used this tool to examine the avian gut microbiome to analyze excreta samples. To our knowledge, our study is the first that analyses the microbiome of different gut segments of broiler chickens at different time intervals by Bugbase. Based on our BugBase analysis, the proportion of potential pathogens in the caecum increased significantly after d 21. Examination of the putative pathogen proportion by other methods [3] did not show such an increase.

### 4.5. Relationship between the Microbiota Composition and IBD Antibody Titre Values

In our study, the serum IBD antibody titre values were measured by an ELISA kit, which measures IgY (IgG) and IgM, but not IgA levels. The majority of immunoglobulins are IgY in the plasma of broiler chickens, which are of maternal origin during the first 10 days [65]. Endogenous production of IgY and IgM in the chick starts around d 10 [66]. The virus from in ovo vaccine Cevac Transmune IBD accumulates in the spleen, and it is neutralised in the blood by maternal antibodies. The decrease in maternal antibody titres during the first three weeks after hatch—which was proved in our experiment as well—due to catabolism of the maternal immunoglobulins, allowing the virus to reach the bursa of the Fabricius and to activate the adaptive immune system. The IBD antibody titre values in serum showed a significant increase from d 21 to d 40 in our experiment, due to an increased synthesis of immunoglobulins. Dietary treatments did not influence serum IBD antibody titre values in our study. Similarly, dietary probiotics did not have an immunomodulatory role and had no significant effects on IBD antibody levels in the experiment of Talebi et al. (2008) [67]. In contrast, other authors reported that probiotic supplementation increased antibody titres against Newcastle disease and IBD virus [68,69,70,71]. Broilact showed a tendency to increase the IBD titre values compared to control group in our experiment at d 7 and 40; however, its effect was not significant.

Antibodies can be secreted on the mucosal surface and into the lumen of the gut, where they can bind antigens and coat microbiota [72]. Moreover, maternal antibodies have been shown to be closely associated with microflora formation and development [73]. The intestine has a high permeability during the first week after hatching, and serum maternal IgY can be transferred from the circulation into the gut mucosa and protect against some enteric pathogens [74,75,76]. Thus, immunoglobulins can influence the composition and development of intestinal microbiota. Janzon et al. (2019) investigated the interactions between the gut microbiome and mucosal IgA, IgM, and IgG in the developing infant gut [72]. To the best of our knowledge, our study is the first that provides information on the relationship between IBD antibody titres in the serum and composition of gut microbiota in chickens during the first 40 days of life. We observed that IBD antibody titre levels negatively correlated with *Lysinibacillus* in both of the investigated periods of IM. As was shown by Huang et al. (2018), the number of this opportunistic pathogen bacterium also increased in *Eimeria*-infected broilers [77]. The opportunistic pathogen *Leuconostoc* bacteria was also associated negatively with IBD antibody titre values at d 7 and 21 in all gut segments. Moreover, a strong negative correlation was observed between the measured immunoglobulins and the opportunistic pathogen bacteria *Pseudomonas*, *Serratia*, *Delftia*, and *Stenotrophomonas* after the third week. Meanwhile, several beneficial bacteria, such as *Bifidobacteria* and *Lactobacillus*, were positively associated with IBD immunoglobulin levels. Immunoglobulin A levels were positively correlated with *Bifidobacteria* OTUs in the faecal samples of infants as well [72].

## 5. Conclusions

According to the results of this trial, we can conclude that using the competitive exclusion product or the synbiotic feed additive at the recommended practical inclusion rate did not affect the production traits of the chickens and had only limited effects on the gut microbiota composition, mostly in the ileal chymus. Some Broilact effects could be detected at d 7, but, interestingly, these changes in the microbiota composition “disappeared” later. The main factors that influence the bacteriota composition are the age of the birds and the place of sampling. This does not mean that pro- and prebiotics are not efficient, but that their effects are probably limited under controlled conditions and in healthy animals. The Bugbase analysis showed that the amount of potentially pathogenic bacteria is the lowest in the IM, but this sample showed the highest variation regarding age. The relative abundance of pathogenic bacteria increased in all gut segments between d 21 and d 40. This result needs further and more detailed investigations and could be interesting for developing special nutritional strategies for the finisher phase of fattening. Significant correlations were found between some bacterial groups and the IBD antibody titre levels. These results suggest that mostly the adaptive immune competence development of the chickens can be supported by the microbiota. In all gut segments, positive correlation was found between the IBD antibody titres and genera *Leuconostoc*, *Bifidobacterium*, *Rombutsia*, and *Turicibacter*. Feed additives that increase the abundance of these genera could be beneficial.

## Figures and Tables

**Table 1:** Effect of dietary treatments on the body weight and daily weight gain of broiler chickens.

Dietary Treatments ^1^	Body Weight (g/bird)				Daily Weight Gain (g/bird)			
	d 0	d 7	d 21	d 40	Starter	Grower	Finisher	Total
C	43	260	1057	2397	217	797	1340	2354
Br	43	256	1065	2398	213	808	1333	2355
Sy	43	260	1073	2441	217	812	1369	2398
Pooled SEM	0.09	2.15	6.65	14.21	2.16	7.17	12.53	15.42
*p*-Values	0.52	0.56	0.89	0.39	0.75	0.69	0.50	0.44

^1^ C—Control, Br—Broilact^®^, Sy—Synbiotic feed additive.

**Table 2:** Effect of dietary treatments on the feed intake and feed conversion ratio of broiler chickens.

Dietary Treatments ^1^	Feed Intake (g/bird)				Feed Conversion Ratio (g/g)			
	Starter	Grower	Finisher	Total	Starter	Grower	Finisher	Total
C	301	1420	2298	4019	1.30	1.58	1.64	1.51
Br	303	1451	2283	4037	1.34	1.59	1.61	1.51
Sy	290	1443	2293	4026	1.26	1.57	1.59	1.48
Pooled SEM	2.87	11.52	42.03	48.47	0.01	0.01	0.03	0.02
*p*-Values	0.11	0.55	0.99	0.99	0.20	0.90	0.83	0.81

^1^ C—Control, Br—Broilact^®^, Sy—Synbiotic feed additive. The production parameters were evaluated with one-way analysis of variance (ANOVA). The differences were considered significant at a level of *p* ≤ 0.05. Data were expressed as means ± SEM.

**Table 3:** Relative abundance of bacterial families in the ileal chymus of broiler chickens as affected by dietary treatments and age (%).

Family	Ileal Chymus						FDR *p*-Values		
	Dietary Treatment	Age of Birds				Mean (Dietary Treatment)			
		d 7	d 14	d 21	d 40		Dietary Treatment	Age	Int.
Lactobacillaceae	C	79.43 ^A^	59.01	63.68	60.68	**65.70**	0.710		0.030
	Br	49.84 ^B^	45.53	69.02	76.02	**60.10**			
	Sy	75.20 ^AB^	56.34	51.58	67.25	**62.59**			
	**Mean (Age)**	**68.16**	**53.62**	**61.43**	**67.99**			0.130	
Enterococcaceae	C	1.74 ^B^	0.16	1.42	1.14	**1.12**	0.510		0.011
	Br	18.17 ^A^	0.32	1.88	0.92	**5.32**			
	Sy	13.76 ^AB^	1.69	2.19	1.76	**4.85**			
	**Mean (Age)**	**11.22 ^a^**	**0.72 ^b^**	**1.83 ^b^**	**1.27 ^b^**			0.001	
Lachnospiraceae	C	6.88	6.68	1.02	0.15	**3.68**	0.640		0.530
	Br	7.55	6.28	0.70	0.19	**3.68**			
	Sy	0.49	1.94	2.99	0.35	**1.44**			
	**Mean (Age)**	**4.97**	**4.96**	**1.57**	**0.23**			0.100	
Enterobacteriaceae	C	0.80	1.76	0.63 ^A^	0.18	**0.85**	0.320		0.079
	Br	9.45	16.68	0.08 ^B^	0.17	**6.60**			
	Sy	0.07	1.62	0.14 ^AB^	0.41	**0.56**			
	**Mean (Age)**	**3.44**	**6.69**	**0.28**	**0.25**			0.065	
Ruminococcaceae	C	4.81	9.04	1.17	0.07	**3.77**	0.830		0.950
	Br	3.51	4.77	0.42	0.10	**2.20**			
	Sy	1.31	5.85	2.23	0.14	**2.38**			
	**Mean (Age)**	**3.21 ^ab^**	**6.55 ^a^**	**1.27 ^b^**	**0.10 ^b^**			0.025	
Mitochondria	C	0.12	0.08	0.02	0.00	**0.05**	0.600		0.450
	Br	0.28	0.27	0.02	0.01	**0.14**			
	Sy	0.51	0.25	0.01	0.00	**0.19**			
	**Mean (Age)**	**0.30 ^a^**	**0.20 ^ab^**	**0.01 ^b^**	**0.00 ^b^**			0.002	
Streptococcaceae	C	1.71	4.66	8.93	12.88	**7.04**	0.600		0.820
	Br	1.26	4.46	5.93	6.84	**4.62**			
	Sy	0.16	4.71	6.50	7.79	**4.79**			
	**Mean (Age)**	**1.04 ^b^**	**4.61 ^b^**	**7.12 ^a^**	**9.17 ^a^**			0.001	
Clostridiaceae_1	C	0.65	0.19	6.20	0.44	**1.87**	0.600		0.420
	Br	0.75	1.35	5.83	0.11	**2.01**			
	Sy	0.65	11.47	6.45	1.26	**4.96**			
	**Mean (Age)**	**0.68**	**4.34**	**6.16**	**0.61**			0.075	
Peptostreptococcaceae	C	1.08	10.57	0.31	8.78	**5.18**	0.860		0.950
	Br	1.08	11.97	0.96	6.64	**5.16**			
	Sy	0.04	7.22	2.17	6.87	**4.07**			
	**Mean (Age)**	**0.73 ^b^**	**9.92 ^a^**	**1.15 ^b^**	**7.43 ^a^**			0.001	
Corynebacteriaceae	C	0.05	0.12	5.63	3.22	**2.25**	0.600		0.770
	Br	0.56	0.07	3.49	2.01	**1.53**			
	Sy	0.09	0.25	8.08	4.49	**3.23**			
	**Mean (Age)**	**0.23 ^b^**	**0.15 ^b^**	**5.73 ^a^**	**3.24 ^ab^**			0.001	
Leuconostocaceae	C	0.05	0.30	2.63	0.75	**0.93**	0.600		0.230
	Br	0.40	0.28	1.69	0.48	**0.71**			
	Sy	0.02	0.21	4.53	0.72	**1.37**			
	**Mean (Age)**	**0.16 ^b^**	**0.26 ^b^**	**2.95 ^a^**	**0.65 ^b^**			0.001	
Staphylococcaceae	C	0.04	0.81	5.72	2.41	**2.24**	0.860		0.880
	Br	0.24	0.19	8.02	0.99	**2.36**			
	Sy	0.08	0.56	7.85	2.57	**2.76**			
	**Mean (Age)**	**0.12 ^b^**	**0.52 ^b^**	**7.20 ^a^**	**1.99 ^b^**			0.001	
Erysipelotrichaceae	C	0.04	0.21	0.19	6.38	**1.71**	0.770		0.360
	Br	0.21	0.13	0.14	3.38	**0.97**			
	Sy	0.04	0.98	0.68	2.04	**0.93**			
	**Mean (Age)**	**0.10 ^b^**	**0.44 ^b^**	**0.34 ^b^**	**3.93 ^a^**			0.001	
Bacillaceae	C	0.01 ^B^	0.03	0.02	0.002 ^B^	0.01 ^B^	0.007		0.370
	Br	0.02 ^B^	0.05	0.01	0.002 ^B^	**0.02** ^**B**^			
	Sy	0.14 ^A^	0.2	0.28	0.04 ^A^	**0.16** ^**A**^			
	**Mean (Age)**	**0.06**	**0.09**	**0.10**	**0.02**			0.230	

Bacterial family differences between groups were assessed using two-way ANOVA test, with Benjamini–Hochberg false discovery rate (FDR) correction. FDR-corrected *p*-values below 0.05 were considered significant. Dietary treatment effects at each sampling days were also compared with one-way ANOVA. The significance of Tukey’s HSD multiple group comparison’s post hoc tests was indicated at *p* ˂ 0.05. a, b: values within the mean (Age) rows with different lowercase letters were significantly different (*p* < 0.05). A, B: values within the mean (d 7, d 40) column with different capital letter superscripts were significantly different (*p* < 0.05). The table shows only those families for which the group average of relative abundance was higher than 1%. “Int.” means the FDR *p*-values of interaction between the two main factors, age, and dietary treatment.

**Table 4:** Relative abundance of bacterial families in the ileal mucosa of broiler chickens as affected by dietary treatments and age (%).

Family	Ileal Mucosa						FDR *p*-Values		
	Dietary Treatment	Age of Birds				Mean (Dietary Treatment)			
		d 7	d 14	d 21	d 40		Dietary Treatment	Day	Int.
Clostridiaceae_1	C	83.77	82.22	70.38	4.26	**60.16**	0.590		0.022
	Br	72.28	78.10	51.62	4.29	**51.57**			
	Sy	42.28	72.84	84.99	12.22	**53.08**			
	**Mean (Age)**	**66.11 ^a^**	**77.72 ^a^**	**69.00 ^a^**	**6.92 ^b^**			0.001	
Peptostreptococcaceae	C	1.06	2.43	1.93	17.75	**5.79**	0.730		0.590
	Br	11.09	4.26	10.64	10.90	**9.22**			
	Sy	12.38	1.62	1.98	18.14	**8.53**			
	**Mean (Age)**	**8.18 ^ab^**	**2.77 ^b^**	**4.85 ^ab^**	**15.60 ^a^**			0.091	
Lactobacillaceae	C	6.48	4.00	11.91 ^AB^	41.35	**15.94**	0.590		0.025
	Br	6.00	5.93	22.64 ^A^	64.41	**24.74**			
	Sy	16.25	9.96	3.90 ^B^	47.03	**19.28**			
	**Mean (Age)**	**9.58 ^b^**	**6.63 ^b^**	**12.82 ^b^**	**50.93 ^a^**			0.001	
Enterococcaceae	C	6.47	0.05	0.45	3.38	**2.59**	0.750		0.950
	Br	3.86	0.06	1.51	1.13	**1.64**			
	Sy	11.95	0.79	0.25	3.62	**4.15**			
	**Mean (Age)**	7.42	0.30	0.74	2.71			0.250	
Lachnospiraceae	C	0.22	1.92	1.76	0.56	**1.11**	0.680		0.680
	Br	1.27	1.99	3.21	1.39	**1.97**			
	Sy	3.41	1.94	1.74	0.32	**1.86**			
	**Mean (Age)**	1.63	1.95	2.24	0.76			0.490	
Burkholderiaceae	C	0.45	0.06	0.19	0.01	**0.18**	0.590		0.110
	Br	1.18	0.08	0.09	0.01	**0.34**			
	Sy	2.67	0.03	0.11	0.00	**0.70**			
	**Mean (Age)**	**1.43 ^a^**	**0.06 ^b^**	**0.13 ^b^**	**0.01 ^b^**			0.002	
Ruminococcaceae	C	0.19	2.82	2.12	0.60	**1.44**	0.990		0.600
	Br	0.83	1.52	2.32	1.54	**1.55**			
	Sy	2.59	2.36	1.17	0.16	**1.57**			
	**Mean (Age)**	**1.20**	**2.24**	**1.87**	**0.77**			0.440	
Pseudomonadaceae	C	0.06	1.98	3.46	0.16	**1.41**	0.980		0.170
	Br	0.25	3.32	1.60	0.10	**1.32**			
	Sy	0.69	3.11	1.32	0.09	**1.30**			
	**Mean (Age)**	**0.33 ^b^**	**2.80 ^a^**	**2.12 ^a^**	**0.12 ^b^**			0.001	
Streptococcaceae	C	0.17	0.50	1.01	14.73	**4.10**	0.590		0.048
	Br	0.20	0.85	1.39	6.76	**2.30**			
	Sy	0.19	2.02	0.51	5.40	**2.03**			
	**Mean (Age)**	**0.19 ^b^**	**1.12 ^b^**	**0.97 ^b^**	**8.96 ^a^**			0.001	
Erysipelotrichaceae	C	0.03	0.09	0.84	10.91	**2.97**	0.670		0.810
	Br	0.01	0.05	0.19	6.06	**1.58**			
	Sy	0.12	0.13	0.24	9.31	**2.45**			
	**Mean (Age)**	**0.06 ^b^**	**0.09 ^b^**	**0.43 ^b^**	**8.76 ^a^**			0.001	
Bacillaceae	C	0.26	0.17	0.17	0.01	**0.15**	0.590		0.077
	Br	0.67	0.09	0.14	0.04	**0.24**			
	Sy	1.66	0.25	0.09	0.01	**0.50**			
	**Mean (Age)**	**0.86 ^a^**	**0.17 ^b^**	**0.14 ^c^**	**0.02 ^d^**			0.003	

Bacterial family differences between groups were assessed using two-way ANOVA test, with Benjamini–Hochberg false discovery rate (FDR) correction. FDR-corrected *p*-values below 0.05 were considered significant. Dietary treatment effects at each sampling days were also compared with one-way ANOVA. The significance of Tukey’s HSD multiple group comparison’s post hoc tests was indicated at *p* ˂ 0.05. a, b, c, d: values within the mean (Day) rows with different lowercase letters were significantly different (*p* < 0.05). A, B: values within the mean (d 7) column with different capital letter superscripts were significantly different (*p* < 0.05). The table shows only those families for which the group average of relative abundance was higher than 1%. “Int.” means the FDR *p*-values of interaction between the two main factors, age, and dietary treatment.

**Table 5:** Relative abundance of bacterial families in the caecum chymus of broiler chickens as affected by dietary treatments and age (%).

Family	Caecum Chymus						FDR *p*-Values		
	Dietary Treatment	Age of Birds				Mean (Dietary Treatment)			
		d 7	d 14	d 21	d 40		Dietary Treatment	Age	Int.
Ruminococcaceae	C	41.49	42.64	36.99	29.84	**37.74**	0.960		0.920
	Br	41.54	47.36	36.87	30.13	**38.98**			
	Sy	39.87	49.55	35.57	29.03	**38.50**			
	**Mean (Age)**	**40.96 ^ab^**	**46.52 ^a^**	**36.48 ^bc^**	**29.67 ^c^**			0.001	
Lachnospiraceae	C	30.35	30.30	21.86	19.39	**25.47**	0.960		0.910
	Br	30.20	27.64	21.22	18.46	**24.38**			
	Sy	33.51	25.03	21.67	18.11	**24.58**			
	**Mean (Age)**	**31.35 ^a^**	**27.65 ^ab^**	**21.58 ^bc^**	**18.65 ^c^**			0.001	
Rikenellaceae	C	0.01	8.90	10.41	5.37	**6.17**	0.970		0.830
	Br	0.06	7.10	8.52	7.33	**5.75**			
	Sy	0.00	8.62	8.15	6.61	**5.84**			
	**Mean (Age)**	**0.02 ^b^**	**8.21 ^a^**	**9.03 ^a^**	**6.44 ^a^**			0.001	
Clostridiales vadinBB60 group	C	10.92	7.53	1.95	2.55	**5.74**	0.960		0.430
	Br	15.68	6.27	2.03	2.32	**6.57**			
	Sy	8.00	8.38	2.82	1.01	**5.05**			
	**Mean (Age)**	**11.53 ^a^**	**7.39 ^a^**	**2.27 ^b^**	**1.96 ^b^**			0.001	
Anaeroplasmataceae	C	4.89	1.87	0.08	0.06	**1.72**	0.960		0.430
	Br	1.31	2.37	0.31	0.08	**1.02**			
	Sy	2.88	1.76	0.08	0.04	**1.19**			
	**Mean (Age)**	**3.03 ^a^**	**2.00 ^ab^**	**0.16 ^b^**	**0.06 ^b^**			0.001	
Lactobacillaceae	C	1.67	3.03	10.95	15.45	**7.78**	0.960		0.020
	Br	0.31	0.91	10.87	21.61	**8.43**			
	Sy	1.74	1.73	8.02	20.85	**8.09**			
	**Mean (Age)**	**1.24 ^c^**	**1.89 ^c^**	**9.95 ^b^**	**19.30 ^a^**			0.001	
Enterobacteriaceae	C	5.46	1.52	0.50	0.16	**1.91**	0.960		0.770
	Br	8.16	3.62	0.38	0.10	**3.07**			
	Sy	10.06	1.94	0.59	0.18	**3.19**			
	**Mean (Age)**	**7.89 ^a^**	**2.36 ^b^**	**0.49 ^b^**	**0.15 ^b^**			0.001	
Christensenellaceae	C	0.20	0.16	1.26	1.69	**0.83**	0.990		0.890
	Br	0.07	0.33	1.07	1.88	**0.84**			
	Sy	0.06	0.43	1.13	1.77	**0.85**			
	**Mean (Age)**	**0.11 ^c^**	**0.31 ^c^**	**1.15 ^b^**	**1.78 ^a^**			0.001	
Erysipelotrichaceae	C	1.50	0.95 ^A^	3.47	6.85 ^A^	**3.19**	0.370		0.150
	Br	1.02	0.52 ^B^	2.97	4.31 ^B^	**2.21**			
	Sy	1.65	0.64 ^AB^	3.39	5.34 ^AB^	**2.75**			
	**Mean (Age)**	**1.39 ^c^**	**0.70 ^c^**	**3.28 ^b^**	**5.50 ^a^**			0.001	
Bacillaceae	C	1.53	0.89	2.07	0.95	**1.36**	0.940		0.980
	Br	0.87	0.72	1.28	0.63	**0.88**			
	Sy	0.82	0.64	1.27	0.85	**0.89**			
	**Mean (Age)**	1.07	0.75	1.54	0.81			0.220	
Peptostreptococcaceae	C	0.08	0.04	1.75	4.81	**1.67**	0.940		0.160
	Br	0.03	0.06	1.23	4.09	**1.35**			
	Sy	0.07	0.07	0.96	5.40	**1.63**			
	**Mean (Age)**	**0.06 ^c^**	**0.06 ^c^**	**1.31 ^b^**	**4.77 ^a^**			0.001	
Akkermansiaceae	C	0.00	0.00	2.59	0.49	**0.77**	0.970		1.000
	Br	0.00	0.01	2.78	0.77	**0.89**			
	Sy	0.00	0.04	2.89	0.82	**0.94**			
	**Mean (Age)**	**0.00 ^b^**	**0.01 ^b^**	**2.75 ^a^**	**0.69 ^b^**			0.001	
Bacteroidaceae	C	0.00	0.71	2.61	6.44	**0.83**	0.960		0.230
	Br	0.00	0.00	6.77	3.93	**1.69**			
	Sy	0.00	0.00	9.45	3.31	**2.36**			
	**Mean (Age)**	**0.00 ^b^**	**0.24 ^b^**	**6.28 ^a^**	**4.56 ^a^**			0.001	
Streptococcaceae	C	0.23	0.14	1.62	1.45	**0.86**	0.960		0.900
	Br	0.03	0.04	1.44	0.69	**0.55**			
	Sy	0.02	0.11	2.09	0.68	**0.72**			
	**Mean (Age)**	**0.09 ^b^**	**0.10 ^b^**	**1.72 ^a^**	**0.94 ^ab^**			0.001	

Bacterial family differences between groups were assessed using two-way ANOVA test, with Benjamini–Hochberg false discovery rate (FDR) correction. FDR-corrected *p*-values below 0.05 were considered significant. Dietary treatment effects at each sampling days were also compared with one-way ANOVA The significance of Tukey’s HSD multiple group comparison’s post hoc tests was indicated at *p* ˂ 0.05. a, b, c: values within the mean (Age) rows with different lowercase letters were significantly different (*p* < 0.05). A, B: values within the mean columns with different capital letter superscripts were significantly different (*p* < 0.05). The table shows only those families for which the group average of relative abundance was higher than 1%. “Int.” means the FDR *p*-values of interaction between the two main factors, age, and dietary treatment.

## Data Availability

All data generated or analysed during this study are included in this published article (and its supplementary information files). Raw sequence data of 16S rRNA metagenomics analysis are deposited in the National Center for Biotechnology Information (NCBI) Sequence Read Archive under the BioProject identifier PRJNA723698.

## Supplementary Materials

The following are available online at https://www.mdpi.com/article/10.3390/vetsci8090187/s1. Figure S1. Microbial profile of Broilact^®^. Figure S2. Rarefaction curves of Species richness. Table S1. Alpha diversity indices of ileal chymus and effects of dietary treatments and age of birds on the microbiome diversity. Table S2. Alpha diversity indices in ileal mucosa and effects of dietary treatments and age of birds on the microbiome diversity. Table S3. Alpha diversity indices in caecum chymus and effects of dietary treatments and age of birds on the microbiome diversity. Table S4. Relative abundance of ileal chymus microbiota at phylum level. Table S5. Relative abundance of ileal mucosa microbiota at phylum level. Table S6. Relative abundance of caecum chymus microbiota at phylum level. Table S7. Relative abundance of ileal chymus microbiota at genus level. Table S8. Relative abundance of ileal mucosa microbiota at genus level. Table S9. Relative abundance of caecum chymus microbiota at genus level. Table S10. Spearman’s correlation between gut microbiota composition and host IBD virus ELISA titres on day 7–21. Table S11. Spearman’s correlation between gut microbiota composition and host Gumboro titer/ parameters on day 21–40. Table S12. Composition and calculated nutrient content of experimental diets (g/kg)^1^.
