# Supplementary material for: Relative Effects of Dietary Administration of a Competitive Exclusion Culture and a Synbiotic Product, Age and Sampling Site on Intestinal Microbiota Maturation in Broiler Chickens
Source: Vet Sci. 2021 Sep 6;8(9):187. doi: 10.3390/vetsci8090187 (PMC8472864; doi:10.3390/vetsci8090187)
Supplement: Supplementary file 1 [file vetsci-08-00187-s001.zip › vetsci-1327597-supplementary.pdf]

Supplementary Material

Dietary Treatments of Competitive Exclusion Culture and Synbiotic Product Influenced Gut Microbiota Maturation in Broiler Chickens

Nikoletta Such [1,†], Valéria Farkas [1,†], Gábor Csitári [1], László Pál [1], Aliz Márton [1], László Menyhárt [2] and Károly Dublecz [1,*]

[1] Institute of Physiology and Nutrition, Hungarian University of Agriculture and Life Sciences, Georgikon Campus, 8360 Keszthely, Hungary; Deák Ferenc Street 16.; farkas.valeria@uni-mate.hu (V.F.); such.nikoletta.amanda@phd.uni-mate.hu (N.S.); csitari.gabor@uni-mate.hu (G. CS.); pal.laszlo@uni-mate.hu (L. P.); marton.aliz@uni-mate.hu (A. M.); dublecz.karoly@uni-mate.hu (K. D.)

[2] Institute of Technology, Hungarian University of Agriculture and Life Sciences, Georgikon Campus, 8360 Keszthely, Hungary; Deák Ferenc Street 16.; menyhart.laszlo@uni-mate.hu (L.M.)

* Correspondence: dublecz.karoly@uni-mate.hu; Tel.: Tel.: +36 30 6418597

† These authors contributed equally to this work.

Table S1. Composition and calculated nutrient content of experimental diets (g/kg) [1].

Ingredients	Starter		Grower		Finisher	
	Basal diet	Sy diet	Basal diet	Sy diet	Basal diet	Sy diet
Maize	430.80	425.35	485.80	480.35	545.30	539.85
Extracted soybean meal	464.00	464.00	412.00	412.00	358.00	358.00
Sunflower oil	56.00	56.00	58.00	58.00	55.00	55.00
Limestone	18.00	18.00	15.00	15.00	14.00	14.00
MCP	16.00	16.00	15.00	15.00	14.00	14.00
Salt	3.00	3.00	3.00	3.00	3.00	3.00
Sodium bicarbonate	1.00	1.00	1.00	1.00	1.00	1.00
L-Lysine-HCl	2.00	2.00	1.00	1.00	1.00	1.00
DL-Methionine	4.00	4.00	3.00	3.00	3.00	3.00
L-Threonine	-	-	1.00	1.00	0.50	0.50
Premix[2]	5.00	5.00	5.00	5.00	5.00	5.00
Phytase[3]	0.10	0.10	0.10	0.10	0.10	0.10
Xylanase[4]	0.10	0.10	0.10	0.10	0.10	0.10
Probiotic supplement [5]	-	0.40	-	0.40	-	0.40
Inulin supplement [6]	-	5.00	-	5.00	-	5.00
Yeast supplement [7]	-	0.05	-	0.05	-	0.05
Sum	*1000.00*	*1000.00*	*1000.00*	*1000.0*	*1000.00*	*1000.00*
Calculated nutrients						
AMEn (MJ/kg) [2]	12.6	12.6	13.0	13.0	13.2	13.2
Crude protein	230.0	229.7	210.0	209.7	190.0	189.7
Crude fibre	28.5	28.4	27.5	27.4	26.5	26.4
Calcium	10.5	10.5	9.0	9.0	8.5	8.5
Phosphorus (available)	5.0	5.0	4.5	4.5	4.2	4.2
dig. Lysine	12.7	12.7	11.0	11.0	9.7	9.7
dig. Methionine	6.5	6.5	5.7	5.7	5.1	5.1
dig. Methionine + Cystine	9.4	9.4	8.4	8.4	7.6	7.6

[1] Basal diet was fed in the C (control) and Br (Broilact®) treatment groups; Sy diet was supplemented with the synbiotic mixture of probiotic, inulin and yeast supplements and fed in the Sy treatment group.

[2]Premix was supplied by UBM Ltd. (Pilisvörösvár, Hungary). The active ingredients in the premix were as follows (per kg of diet): retinyl acetate – 5.0 mg, cholecalciferol – 130 µg, dl-alpha-tocopherol-acetate – 91 mg, menadione – 2.2 mg, thiamin – 4.5 mg, riboflavin – 10.5 mg, pyridoxin HCL – 7.5 mg, cyanocobalamin – 80 µg, niacin – 41.5 mg, pantothenic acid – 15 mg, folic acid – 1.3 mg, biotin – 150 µg, betaine – 670 mg, Ronozyme® NP – 150 mg, monensin-Na – 110 mg (only grower), narasin – 50 mg (only starter), nicarbazin – 50 mg (only starter), antioxidant – 25 mg, Zn (as $ZnSO_4{\cdot}H_2O$) – 125 mg, Cu (as $CuSO_4{\cdot}5H_2O$) – 20 mg, Fe (as $FeSO_4{\cdot}H_2O$) – 75 mg, Mn (as MnO) – 125 mg, I (as KI) – 1.35 mg, Se (as Na_2SeO_3) – 270 µg;

[3] Quantum Blue® 5G (AB Vista, Marlborough, Wiltshire, SN8 4AN, United Kingdom)

[4] Econase® XT 25P (AB Vista, Marlborough, Wiltshire, SN8 4AN, United Kingdom)

[5] GalliPro® 200 (*Bacillus subtilis*, DSM17299 bacterial strain; 1.6×10^6 CFU/g, Biochem Ltd., Küstermeyerstrasse 16. 49393 Lohne, Germany)

[6] Orafti® HSI (Beneo Ltd., Aandorenstraat 1, B. 3300 Tienen, Belgium)

[7] Levucell® SB20 (*Saccharomyces cerevisiae boulardii*, 2×10^{10} CFU/g, Lallemand Ltd., Ottakringer Str. 89, A-1160 Vienna, Austria)

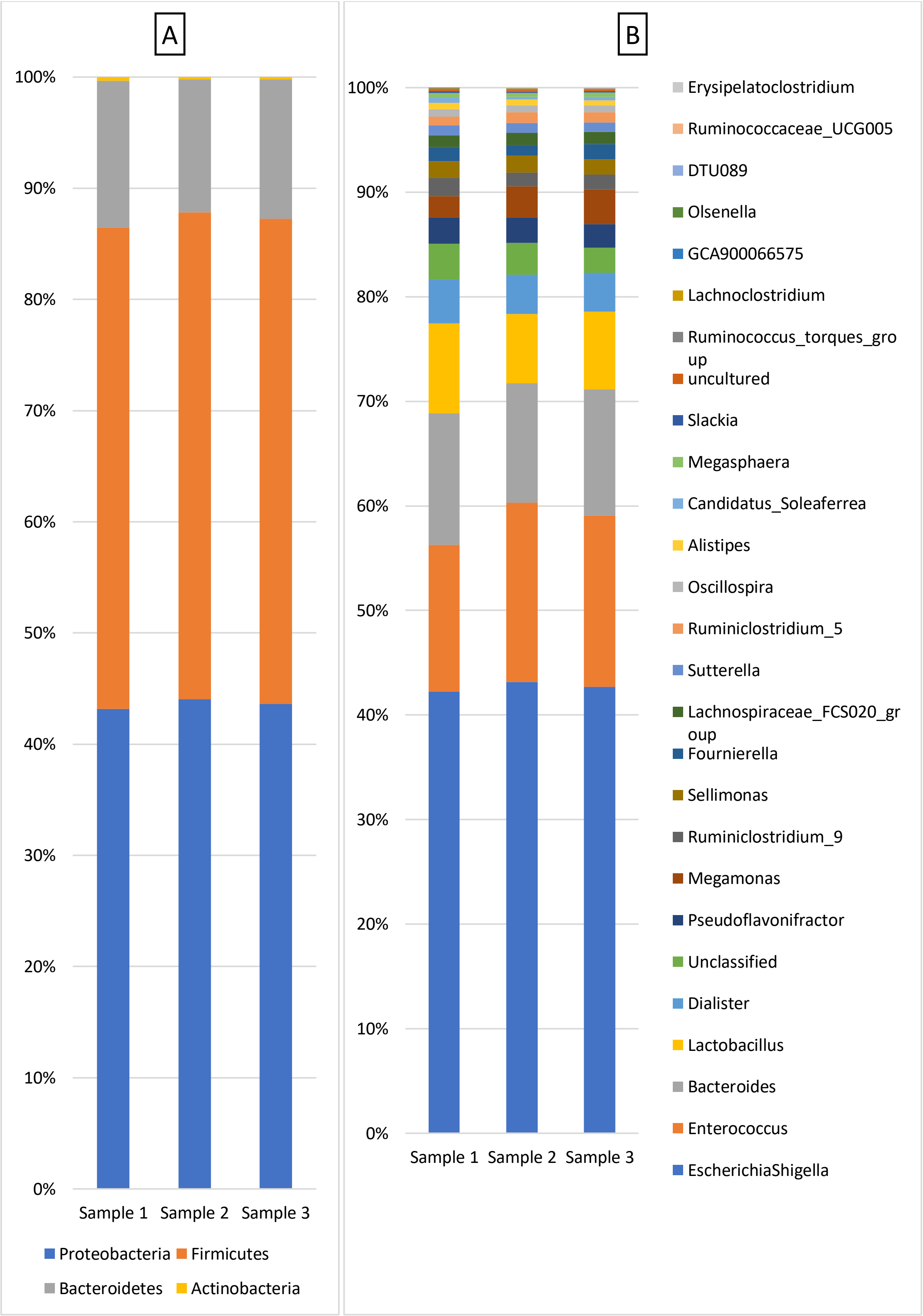

Figure S1. Microbial profile of Broilact®. Stacked column plots show the percent mean relative abundance of bacterial phyla (**A**) and genera (**B**).

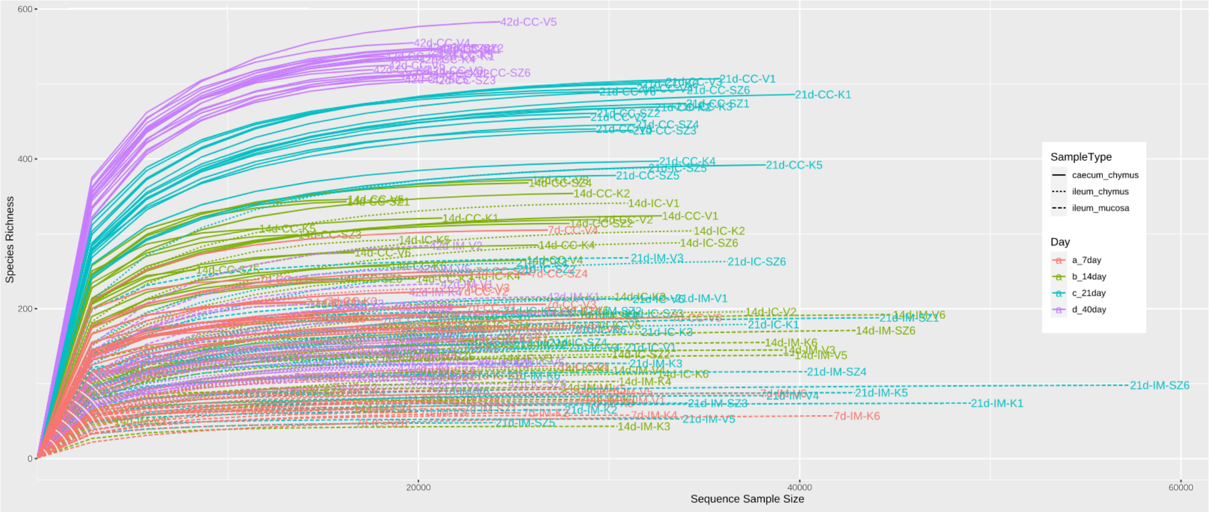

Figure S2. Rarefaction curves of Species richness. The observed metrics were number of Operational Taxonomic Units (OTUs). Each colored line represents the microbiota of a single chicken sample. Line color based on age of chicken; line type based on sample type (dashed: ileum mucosa; scored: ileum chymus; straight: caecum chymus). Sequence data were rarefied to a depth of 1000 sequences per sample.

Table S2. Alpha diversity indices of ileum chymus and effects of dietary treatments (C – Control, Br – Broilact®, Sy – Synbiotic feed additive) and age of birds on the microbiota diversity.

Diversity Indices	Ileum Chymus						*p*-Value		
	Dietary Treatment	Age of Birds				Mean (Dietary Treatment)	Dietary Treatment	Age	Interaction
		d 7	d 14	d 21	d 40				
Chao1	C	114.20^{B}	215.56	195.39	128.79	**163.48**	0.853		0.140
	Br	175.87^{A}	200.56	173.95	130.69	**170.27**			
	Sy	122.48^{AB}	188.36	234.62	138.96	**171.11**			
	**Mean (Age)**	**137.52^{a}**	**201.5^{b}**	**201.3^{b}**	**132.8^{a}**			0.001	
Shannon	C	1.94^{B}	2.60	3.02	2.95	**2.63**	0.678		0.011
	Br	2.96^{A}	2.61	2.86	2.46	**2.73**			
	Sy	2.37^{AB}	2.54	3.23	2.80	**2.73**			
	**Mean (Age)**	**2.42^{a}**	**2.58^{a}**	**3.04^{b}**	**2.74^{ab}**			0.002	
Simpson	C	0.69 ^{B}	0.79	0.89	0.90	**0.82**	0.501		0.024
	Br	0.89 ^{A}	0.82	0.87	0.81	**0.85**			
	Sy	0.81 ^{AB}	0.79	0.91	0.86	**0.84**			
	**Mean (Age)**	**0.80 ^{a}**	**0.80 ^{a}**	**0.89 ^{b}**	**0.86 ^{ab}**			0.009	

Averages of the columns with different capital letter superscripts and values within the mean rows with different lowercase letters are significantly different ($p < 0.05$).

Table S3. Alpha diversity indices in ileum mucosa and effects of dietary treatments and age of birds on the microbiota diversity.

Diversity Indices	Ileum Mucosa						*p*-Value		
	Dietary Treatment	Age of Birds				Mean (Dietary Treatment)	Dietary Treatment	Age	Interaction
		d 7	d 14	d 21	d 40				
Chao1	C	73.34	113.38	99.91	186.95 ^{A}	**118.39**	0.051		0.060
	Br	75.32	123.31	143.84	226.45 ^{A}	**142.23**			
	Sy	80.40	122.56	120.66	128.18 ^{B}	**112.95**			
	**Mean (Age)**	**76.36** ^{a}	**119.75** ^{a}	**121.47** ^{b}	**180.53** ^{c}			0.001	
Shannon	C	0.73	1.09	1.58	3.02 ^{A}	**1.60**	0.886		0.036
	Br	0.89	1.22	1.91	2.73 ^{AB}	**1.69**			
	Sy	1.73	1.45	0.96	2.62 ^{B}	**1.69**			
	**Mean (Age)**	**1.12** ^{a}	**1.25** ^{a}	**1.48** ^{a}	**2.79** ^{b}			0.001	
Simpson	C	0.27	0.31	0.45	0.89	**0.48**	0.643		0.037
	Br	0.29	0.38	0.57	0.85	**0.52**			
	Sy	0.55	0.44	0.27	0.86	**0.53**			
	**Mean (Age)**	**0.37** ^{a}	**0.38** ^{a}	**0.43** ^{a}	**0.87** ^{b}			0.001	

Averages of the columns with different capital letter superscripts and values within the mean rows with different lowercase letters are significantly different ($p < 0.05$).

Table S4. Alpha diversity indices in caecum chymus and effects of dietary treatments and age of birds on the microbiota diversity.

Diversity Indices	Caecum Chymus						*p*-Value		
	Dietary Treatment	Age of Birds				Mean (Dietary Treatment)	Dietary Treatment	Age	Interaction
		d 7	d 14	d 21	d 40				
Chao1	C	201.74	294.96	453.91	530.71	**370.33**	0.124		0.983
	Br	216.81	317.95	483.32	542.08	**390.04**			
	Sy	207.32	303.37	449.33	529.75	**372.44**			
	**Mean (Age)**	**208.63** ^{a}	**305.43** ^{b}	**462.19** ^{c}	**534.18** ^{d}			0.001	
Shannon	C	3.79	4.18	4.34	4.77	**4.27**	0.119		0.372
	Br	3.51	4.12	4.34	4.59	**4.14**			
	Sy	3.82	4.14	4.24	4.70	**4.23**			
	**Mean (Age)**	**3.71** ^{a}	**4.15** ^{b}	**4.31** ^{b}	**4.69** ^{c}			0.001	
Simpson	C	0.948	0.963	0.962	0.978	**0.963** ^{A}	0.024		0.232
	Br	0.930	0.962	0.955	0.966	**0.953** ^{B}			
	Sy	0.952	0.960	0.950	0.977	**0.960** ^{AB}			
	**Mean (Age)**	**0.943** ^{a}	**0.962** ^{bc}	**0.955** ^{b}	**0.974** ^{c}			0.001	

Averages of the columns with different capital letter superscripts and values within the mean rows with different lowercase letters are significantly different ($p < 0.05$).

Table S5. Relative abundance of Ileum chymus microbiota at phylum level.

Phylum	Ileum Chymus (%)						FDR *p*-Value		
	Dietary Treatment	Age of Birds				Mean (Dietary Treatment)	Dietary Treatment	Age	Interaction
		d 7	d 14	d 21	d 40				
Firmicutes	C	96.49 ^{A}	91.68	92.65	94.80	**93.91**	0.180		0.032
	Br	83.95 ^{B}	75.59	95.09	96.31	**87.74**			
	Sy	92.01 ^{AB}	91.39	89.72	92.66	**91.45**			
	**Mean (Age)**	**90.82 ^{ab}**	**86.22 ^{b}**	**92.49 ^{ab}**	**94.59 ^{a}**			0.055	
Proteobacteria	C	1.48	2.08	0.73 ^{A}	0.19	**1.12**	0.060		0.083
	Br	10.85	17.07	0.15 ^{B}	0.20	**7.07**			
	Sy	2.09	2.15	0.18 ^{AB}	0.43	**1.21**			
	**Mean (Age)**	**4.81 ^{ab}**	**7.10 ^{a}**	**0.35 ^{b}**	**0.27 ^{b}**			0.026	
Cyanobacteria	C	1.93 ^{B}	3.32	0.12 ^{B}	0.33	**1.43**	0.110		0.390
	Br	4.35 ^{AB}	5.74	0.08 ^{AB}	0.11	**2.57**			
	Sy	5.49 ^{A}	5.39	0.18 ^{A}	0.24	**3.16**			
	**Mean (Age)**	**3.92 ^{a}**	**4.82 ^{a}**	**0.13 ^{b}**	**0.23 ^{b}**			0.001	
Actinobacteria	C	0.05	0.15	6.08	4.33	**2.65**	0.540		0.830
	Br	0.65	0.09	4.44	3.11	**2.07**			
	Sy	0.12	0.26	8.74	5.05	**3.54**			
	**Mean (Age)**	**0.27 ^{b}**	**0.17 ^{b}**	**6.42 ^{a}**	**4.16 ^{a}**			0.001	
Bacteroidetes	C	0.03 ^{B}	2.53	0.15	0.00	**0.68**	0.360		0.008
	Br	0.06 ^{AB}	0.19	0.12	0.04	**0.10**			
	Sy	0.12 ^{A}	0.39	1.08	0.01	**0.40**			
	**Mean (Age)**	**0.07 ^{b}**	**1.04 ^{a}**	**0.45 ^{ab}**	**0.02 ^{b}**			0.024	
Patescibacteria	C	0.00	0.20	0.08	0.34	**0.16**	0.360		0.036
	Br	0.07	1.29	0.06	0.23	**0.41**			
	Sy	0.12	0.21	0.08	0.34	**0.19**			
	**Mean (Age)**	**0.06 ^{b}**	**0.57 ^{a}**	**0.07 ^{b}**	**0.30 ^{ab}**			0.026	
Verrucomicrobia	C	0.00	0.00	0.17	0.00	**0.04**	0.640		0.820
	Br	0.00	0.00	0.06	0.00	**0.02**			
	Sy	0.00	0.00	0.16	0.00	**0.04**			
	**Mean (Age)**	**0.00 ^{b}**	**0.001 ^{b}**	**0.13 ^{a}**	**0.001 ^{b}**			0.003	
Tenericutes	C	0.01	0.03	0.02	0.00	**0.02**	0.610		0.650
	Br	0.03	0.02	0.00	0.00	**0.01**			
	Sy	0.00	0.01	0.01	0.00	**0.01**			
	**Mean (Age)**	**0.01**	**0.02**	**0.01**	**0.00**			0.260	
Armatimonadetes	C	0.01	0.00	0.00	0.00	**0.00**	0.410		0.210
	Br	0.04	0.00	0.00	0.00	**0.01**			
	Sy	0.04	0.00	0.00	0.00	**0.01**			
	**Mean (Age)**	**0.03 ^{a}**	**0.00 ^{b}**	**0.00 ^{b}**	**0.00 ^{b}**			0.001	
Chloroflexi	C	0.00	0.00	0.00	0.00	**0.00**	0.490		0.430
	Br	0.00	0.00	0.00	0.00	**0.00**			
	Sy	0.00	0.00	0.00	0.00	**0.00**			
	**Mean (Age)**	0.00	0.00	0.00	0.00			0.400	

Averages of the columns with different capital letter superscripts and values within the mean rows with different lowercase letters are significantly different ($p < 0.05$).

Table S6. Relative abundance of Ileum mucosa microbiota at phylum level.

Phylum	Ileum Mucosa (%)						FDR *p*-Value		
	Dietary Treatment	Age of Birds				Mean (Dietary Treatment)	Dietary Treatment	Age	Interaction
		d 7	d 14	d 21	d 40				
Firmicutes	C	98.77 ^{A}	95.35	92.57	96.11	**95.70**	0.710		0.042
	Br	96.30 ^{AB}	93.21	95.09	97.72	**95.58**			
	Sy	91.84 ^{B}	92.72	96.13	97.86	**94.64**			
	**Mean (Age)**	**95.64**	**93.76**	**94.60**	**97.23**			0.140	
Proteobacteria	C	0.90 ^{B}	3.19	5.83	1.56	**2.87**	0.860		0.021
	Br	2.94 ^{AB}	5.30	2.78	0.52	**2.89**			
	Sy	6.03 ^{A}	5.07	2.15	0.61	**3.47**			
	**Mean (Age)**	**3.29 ^{ab}**	**4.52 ^{a}**	**3.59 ^{ab}**	**0.90 ^{b}**			0.030	
Actinobacteria	C	0.23 ^{B}	0.34	0.62	1.80	**0.75**	0.710		0.120
	Br	0.60 ^{AB}	0.63	0.32	0.92	**0.62**			
	Sy	1.26 ^{A}	0.58	0.47	1.10	**0.85**			
	**Mean (Age)**	**0.70 ^{ab}**	**0.52 ^{b}**	**0.47 ^{b}**	**1.27 ^{a}**			0.060	
Bacteroidetes	C	0.02	0.65	0.44	0.14	**0.31**	0.880		0.540
	Br	0.01	0.13	1.21	0.64	**0.50**			
	Sy	0.00	0.20	1.02	0.07	**0.32**			
	**Mean (Age)**	**0.01**	**0.33**	**0.89**	**0.28**			0.140	
Cyanobacteria	C	0.08 ^{B}	0.35	0.00	0.14	**0.14**	0.120		0.041
	Br	0.14 ^{B}	0.58	0.14	0.08	**0.24**			
	Sy	0.85 ^{A}	1.00	0.01	0.10	**0.49**			
	**Mean (Age)**	**0.36 ^{ab}**	**0.64 ^{a}**	**0.05 ^{b}**	**0.11 ^{b}**			0.001	
Patescibacteria	C	0.00	0.02	0.00	0.23	**0.06**	0.710		0.630
	Br	0.00	0.11	0.04	0.10	**0.06**			
	Sy	0.00	0.35	0.00	0.25	**0.15**			
	**Mean (Age)**	**0.00**	**0.16**	**0.01**	**0.19**			0.140	
Verrucomicrobia	C	0.00	0.00	0.33	0.01	**0.09**	0.880		0.990
	Br	0.00	0.00	0.36	0.01	**0.09**			
	Sy	0.00	0.01	0.19	0.00	**0.05**			
	**Mean (Age)**	**0.00 ^{b}**	**0.00 ^{b}**	**0.29 ^{a}**	**0.01 ^{b}**			0.060	
Tenericutes	C	0.00	0.04	0.16	0.01	**0.05**	0.710		0.780
	Br	0.00	0.04	0.05	0.01	**0.03**			
	Sy	0.00	0.07	0.02	0.00	**0.02**			
	**Mean (Age)**	**0.00**	**0.05**	**0.08**	**0.01**			0.210	
Deinococcus_Thermus	C	0.00	0.00	0.04	0.00	**0.01**	0.710		0.540
	Br	0.00	0.00	0.00	0.00	**0.00**			
	Sy	0.00	0.00	0.01	0.01	**0.01**			
	**Mean (Age)**	**0.00**	**0.00**	**0.02**	**0.00**			0.400	
Chloroflexi	C	0.00	0.01	0.00	0.00	**0.00**	0.790		0.610
	Br	0.01	0.00	0.00	0.00	**0.00**			
	Sy	0.00	0.00	0.00	0.00	**0.00**			
	**Mean (Age)**	**0.00**	**0.00**	**0.00**	**0.00**			0.210	

Averages of the columns with different capital letter superscripts and values within the mean rows with different lowercase letters are significantly different ($p < 0.05$).

Table S7. Relative abundance of caecum chymus microbiota at phylum level.

Phylum	Caecum Chymus (%)						FDR *p*-Value		
	Dietary Treatment	Age of Birds				Mean (Dietary Treatment)			
		d 7	d 14	d 21	d 40		Dietary Treatment	Day	Interaction
Firmicutes	C	88.60	86.21	83.19	85.66	**85.92**	0.940		0.740
	Br	90.19	84.49	80.66	86.13	**85.37**			
	Sy	86.47	87.25	77.98	87.05	**84.69**			
	**Mean (Age)**	**88.42** ^{a}	**85.98** ^{a}	**80.61** ^{b}	**86.28** ^{a}			0.003	
Proteobacteria	C	5.50	1.65	0.59	1.02	**2.19**	0.940		0.730
	Br	8.22	3.88	0.46	0.87	**3.36**			
	Sy	10.22	1.96	0.73	1.05	**3.49**			
	**Mean (Age)**	**7.98** ^{a}	**2.50** ^{b}	**0.59** ^{b}	**0.98** ^{b}			0.001	
Bacteroidetes	C	0.01	9.61	13.02	11.82	**8.62**	0.940		0.700
	Br	0.06	7.10	15.29	11.26	**8.43**			
	Sy	0.00	8.62	17.60	9.92	**9.04**			
	**Mean (Age)**	**0.02** ^{c}	**8.44** ^{b}	**15.30** ^{a}	**11.00** ^{b}			0.001	
Tenericutes	C	5.86	2.01	0.34	0.33	**2.14**	0.940		0.160
	Br	1.49	2.53	0.62	0.33	**1.24**			
	Sy	3.29	1.92	0.46	0.28	**1.49**			
	**Mean (Age)**	**3.55** ^{a}	**2.15** ^{ab}	**0.47** ^{b}	**0.31** ^{b}			0.001	
Cyanobacteria	C	0.00	0.45	0.18	0.59	**0.31**	0.940		0.280
	Br	0.00	1.93	0.08	0.56	**0.64**			
	Sy	0.00	0.18	0.20	0.67	**0.26**			
	**Mean (Age)**	**0.00**	**0.85**	**0.15**	**0.61**			0.073	
Actinobacteria	C	0.03	0.07	0.10	0.07	**0.07**	0.940		0.230
	Br	0.03	0.05	0.11	0.07	**0.07**			
	Sy	0.03	0.04	0.15	0.19	**0.10**			
	**Mean (Age)**	**0.03** ^{b}	**0.05** ^{ab}	**0.12** ^{a}	**0.11** ^{a}			0.002	
Verrucomicrobia	C	0.00	0.00	2.59	0.49	**0.77**	0.940		1.000
	Br	0.00	0.01	2.78	0.77	**0.89**			
	Sy	0.00	0.04	2.89	0.82	**0.94**			
	**Mean (Age)**	**0.00** ^{b}	**0.02** ^{b}	**2.75** ^{a}	**0.69** ^{b}			0.001	
Patescibacteria	C	0.00	0.00	0.00	0.02	**0.01**	0.940		0.580
	Br	0.00	0.00	0.00	0.01	**0.00**			
	Sy	0.00	0.00	0.00	0.02	**0.01**			
	**Mean (Age)**	**0.00** ^{b}	**0.00** ^{b}	**0.00** ^{b}	**0.02** ^{a}			0.001	

Averages of the columns with different capital letter superscripts and values within the mean rows with different lowercase letters are significantly different ($p < 0.05$).

Table S8. Relative abundance of Ileum chymus microbiota at genus level.

Genus	Ileum Chymus (%)						FDR p-Value		
	Dietary Treatment	Age of Birds				Mean (Dietary Treatment)	Dietary Treatment	Age	Interaction
		d 7	d 14	d 21	d 40				
Lactobacillus	C	79.43 A	61.30	63.68	60.68	**66.27**	0.820		0.030
	Br	49.84 B	45.53	69.02	76.02	**60.10**			
	Sy	75.19 AB	56.34	51.58	67.25	**62.59**			
	**Mean (Age)**	**68.15**	**54.39**	**61.43**	**67.99**			0.150	
Enterococcus	C	1.74 B	0.47	1.42	1.14	**1.19**	0.800		0.011
	Br	18.17 A	0.32	1.88	0.91	**5.32**			
	Sy	13.76 AB	1.69	2.19	1.74	**4.84**			
	**Mean (Age)**	**11.22 a**	**0.82 b**	**1.83 b**	**1.26 b**			0.001	
Escherichia-Shigella	C	0.80	1.49	0.58 A	0.17	**0.76**	0.710		0.079
	Br	9.44	16.66	0.06 B	0.16	**6.58**			
	Sy	0.07	1.55	0.13 AB	0.30	**0.51**			
	**Mean (Age)**	**3.44**	**6.57**	**0.26**	**0.21**			0.081	
Subdoligranulum	C	3.19	0.14	0.07	0.01	**0.85**	0.820		0.730
	Br	2.48	0.23	0.03	0.01	**0.69**			
	Sy	0.11	0.31	0.14	0.06	**0.15**			
	**Mean (Age)**	**1.92**	**0.23**	**0.08**	**0.03**			0.170	
Streptococcus	C	1.71	3.65	2.90	11.95	**5.05**	0.800		0.830
	Br	1.26	0.80	2.68	6.39	**2.78**			
	Sy	0.16	1.54	2.96	7.24	**2.97**			
	**Mean (Age)**	**1.04 b**	**2.00 b**	**2.84 b**	**8.53 a**			0.001	
Clostridium sensu stricto 1	C	0.44	0.31	6.11	0.29	**1.79**	0.960		1.000
	Br	0.14	0.08	5.30	0.35	**1.47**			
	Sy	0.34	0.30	5.50	0.10	**1.56**			
	**Mean (Age)**	**0.30 b**	**0.23 b**	**5.64 a**	**0.25 b**			0.001	
Corynebacterium 1	C	0.09	0.25	8.08	4.49	**3.23**	0.800		0.770
	Br	0.05	0.11	5.63	3.22	**2.25**			
	Sy	0.56	0.07	3.49	2.01	**1.53**			
	**Mean (Age)**	**0.23 b**	**0.14 b**	**5.73 a**	**3.24 ab**			0.001	
Candidatus-Arthromitus	C	0.09	11.12	0.13	0.97	**3.08**	0.800		0.240
	Br	0.47	0.58	0.58	0.09	**0.43**			
	Sy	0.11	0.99	0.14	0.01	**0.31**			
	**Mean (Age)**	**0.22**	**4.23**	**0.28**	**0.36**			0.230	
Staphylococcus	C	0.07	0.54	7.31	1.25	**2.29**	0.940		0.860
	Br	0.04	0.71	5.24	1.60	**1.90**			
	Sy	0.20	0.19	7.75	0.70	**2.21**			
	**Mean (Age)**	**0.11 b**	**0.48 b**	**6.76 a**	**1.18 b**			0.001	
Butyricicoccus	C	0.04	0.96	0.10	0.00	**0.28**	0.820		0.820
	Br	0.27	1.56	0.07	0.00	**0.48**			
	Sy	0.16	0.49	0.01	0.00	**0.17**			
	**Mean (Age)**	**0.16 b**	**1.00 a**	**0.06 b**	**0.00 b**			0.018	
Weissella	C	0.02	0.20	3.03	0.57	**0.95**	0.800		0.420
	Br	0.05	0.29	1.74	0.23	**0.58**			
	Sy	0.40	0.28	0.63	0.11	**0.36**			
	**Mean (Age)**	**0.16 b**	**0.26 b**	**1.80 a**	**0.30 b**			0.014	
Romboutsia	C	0.02	3.30	1.85	3.06	**2.06**	0.950		0.640
	Br	0.01	2.18	0.24	7.76	**2.55**			
	Sy	0.11	0.86	0.78	6.41	**2.04**			
	**Mean (Age)**	**0.05 b**	**2.11 b**	**0.96 b**	**5.74 a**			0.010	
Turicibacter	C	0.00	0.90	0.54	2.01	**0.86**	0.820		0.360
	Br	0.00	0.01	0.15	6.38	**1.64**			
	Sy	0.00	0.02	0.12	3.37	**0.88**			
	**Mean (Age)**	**0.00 b**	**0.31 b**	**0.27 b**	**3.92 a**			0.001	
Lactococcus	C	0.00	3.17	3.54	0.55	**1.82**	0.990		0.160
	Br	0.00	0.59	6.03	0.93	**1.89**			

	Sy	0.00	3.66	3.26	0.45	**1.84**			
	**Mean (Age)**	**0.00** [b]	**2.48** [a]	**4.28** [a]	**0.64** [b]			0.001	
Leuconostoc	C	0.00	0.01	1.50	0.16	**0.42**	0.920		0.200
	Br	0.00	0.00	0.89	0.52	**0.35**			
	Sy	0.00	0.00	1.06	0.37	**0.36**			
	**Mean (Age)**	**0.00** [b]	**0.00** [b]	**1.15** [a]	**0.35** [b]	**66.27**		0.001	
Bacillus	C	0.01 [B]	0.03	0.02	0.002 [B]	**0.01**	0.019		0.360
	Br	0.01 [B]	0.05	0.01	0.002 [B]	**0.02**			
	Sy	0.12 [A]	0.20	0.28	0.043 [A]	**0.16**			
	**Mean (Age)**	**4.67**	**9.33**	**10.33**	**1.57**			0.250	
Ruminococcaceae UCG-014	C	0.23	1.12	0.25	0.01	**0.48**	0.780		0.860
	Br	0.09	1.04	0.80	0.01	**0.40**			
	Sy	1.08	1.45	0.57	0.02	**0.78**			
	**Mean (Age)**	**0.46** [ab]	**1.20** [a]	**0.54** [ab]	**0.01** [b]			0.047	

Averages of the columns with different capital letter superscripts and values within the mean rows with different lowercase letters are significantly different ($p < 0.05$).

Table S9. Relative abundance of Ileum mucosa microbiota at genus level.

Genus	Ileum Mucosa (%)						FDR p-Value		
	Dietary Treatment	Age of Birds				Mean (Dietary Treatment)	Dietary Treatment	Age	Interaction
		d 7	d 14	d 21	d 40				
Candidatus Arthromitus	C	83.76	82.20	70.15	3.51	**59.90**	0.660		0.021
	Br	72.26	78.03	51.15	3.93	**51.34**			
	Sy	42.19	72.73	84.89	11.62	**52.86**			
	**Mean (Age)**	**66.07** [a]	**77.65** [a]	**68.73** [a]	**6.35** [b]			0.001	
Lactobacillus	C	6.48	3.94	11.91 [AB]	41.35	**15.92**	0.660		0.025
	Br	6.00	5.93	22.64 [A]	64.41	**24.74**			
	Sy	16.25	9.96	3.90 [B]	47.03	**19.28**			
	**Mean (Age)**	**9.58** [b]	**6.61** [b]	**12.82** [b]	**50.93** [a]			0.001	
Enterococcus	C	6.47	0.05	0.45	3.37	**2.58**	0.770		0.950
	Br	3.86	0.06	1.51	1.13	**1.64**			
	Sy	11.95	0.79	0.25	3.61	**4.15**			
	**Mean (Age)**	**7.42**	**0.30**	**0.74**	**2.70**			0.320	
Delftia	C	0.35	0.01	0.15	0.01	**0.13**	0.660		0.110
	Br	1.08	0.00	0.06	0.00	**0.29**			
	Sy	2.46	0.00	0.07	0.00	**0.63**			
	**Mean (Age)**	**1.30** [a]	**0.00** [b]	**0.10** [b]	**0.00** [b]			0.001	
Romboutsia	C	0.05	1.32	0.80	10.33	**0.04**	0.990		1.000
	Br	0.13	1.84	0.91	9.71	**0.11**			
	Sy	1.27	0.81	0.47	10.84	**0.25**			
	**Mean (Age)**	**0.48** [b]	**1.32** [b]	**0.73** [b]	**10.29** [a]			0.001	
Pseudomonas	C	0.06	1.98	3.46	0.16	**3.12**	0.980		0.170
	Br	0.25	3.32	1.60	0.10	**3.15**			
	Sy	0.69	3.11	1.32	0.09	**3.35**			
	**Mean (Age)**	**0.33** [b]	**2.80** [a]	**2.12** [a]	**0.12** [b]			0.001	
Streptococcus	C	0.17	0.19	0.78	14.30	**1.41**	0.660		0.074
	Br	0.20	0.24	1.20	6.55	**1.32**			
	Sy	0.19	0.59	0.44	5.26	**1.30**			
	**Mean (Age)**	**0.19** [b]	**0.34** [b]	**0.81** [b]	**8.70** [a]			0.001	
Turicibacter	C	0.01	0.00	0.81	10.88	**3.86**	0.700		0.800
	Br	0.00	0.00	0.05	5.97	**2.05**			
	Sy	0.01	0.12	0.17	9.30	**1.62**			
	**Mean (Age)**	**0.01** [b]	**0.04** [b]	**0.35** [b]	**8.72** [a]			0.001	
Lysinibacillus	C	0.001	0.001	0.441	0.003	**0.011**	0.600		0.002
	Br	0.001	0.001	0.139	0.001	**0.035**			
	Sy	0.005	0.001	0.137	0.001	**0.036**			
	**Mean (Age)**	**0.002** [b]	**0.001** [b]	**0.239** [a]	**0.001** [a]			0.001	
Ruminococcaceae UCG-008	C	0.001	0.001	0.001	0.024 [B]	**0.006**	0.001		0.001
	Br	0.001	0.001	0.002	0.089 [A]	**0.023**			
	Sy	0.001	0.001	0.001	0.009 [B]	**0.002**			
	**Mean (Age)**	**0.001** [b]	**0.001** [b]	**0.001** [b]	**0.041** [a]			0.001	
Bacillus	C	0.061	0.154	0.166	0.008	**0.097**	0.660		0.220
	Br	0.085	0.064	0.136	0.042	**0.082**			
	Sy	0.243	0.210	0.094	0.012	**0.140**			
	**Mean (Age)**	**0.130**	**0.143**	**0.132**	**0.021**			0.110	

Averages of the columns with different capital letter superscripts and values within the mean rows with different lowercase letters are significantly different ($p < 0.05$).

Table S10. Relative abundance of caecum chymus microbiota at genus level.

Genus	Caecum Chymus (%)						FDR *p*-Value		
	Dietary Treatment	Age of Birds				Mean (Dietary Treatment)	Dietary Treatment	Age	Interaction
		d 7	d 14	d 21	d 40				
Escherichia-Shigella	C	5.46	1.52	0.49	0.16	**1.91**	0.910		0.770
	Br	8.15	3.62	0.37	0.10	**3.06**			
	Sy	10.06	1.94	0.59	0.18	**3.19**			
	**Mean (Age)**	**7.89** [a]	**2.36** [b]	**0.48** [b]	**0.15** [b]			0.001	
Subdoligranulum	C	8.69	1.68	1.79	1.12	**3.32**	0.890		0.430
	Br	10.32	1.23	0.99	0.80	**3.33**			
	Sy	1.43	0.88	1.56	1.09	**1.24**			
	**Mean (Age)**	**6.81** [a]	**1.26** [b]	**1.45** [b]	**1.00** [b]			0.015	
Butyricicoccus	C	4.85	2.86	2.56	1.31	**2.89**	0.890		0.370
	Br	5.01	3.50	2.25	0.62	**2.84**			
	Sy	5.36	7.24	2.94	0.95	**4.12**			
	**Mean (Age)**	**5.07** [a]	**4.53** [ab]	**2.58** [bc]	**0.96** [c]			0.001	
Ruminococcaceae UCG-014	C	3.84	7.01	4.02	5.08	**1.92**	0.910		0.440
	Br	2.79	4.54	4.98	5.64	**1.99**			
	Sy	3.49	4.68	4.33	5.39	**1.76**			
	**Mean (Age)**	**3.37** [b]	**5.41** [a]	**4.44** [ab]	**5.37** [a]			0.022	
Anaeroplasma	C	4.89	1.87	0.08	0.06	**4.99**	0.910		0.430
	Br	1.31	2.37	0.31	0.08	**4.49**			
	Sy	2.88	1.76	0.08	0.04	**4.47**			
	**Mean (Age)**	**3.03** [a]	**2.00** [ab]	**0.16** [b]	**0.06** [b]			0.001	
Ruminococcus torques group	C	2.23	7.99	1.35	1.90	**1.72**	0.910		0.390
	Br	3.14	3.19	1.79	1.73	**1.02**			
	Sy	2.90	5.00	1.88	1.44	**1.19**			
	**Mean (Age)**	**2.75** [ab]	**5.40** [b]	**1.67** [b]	**1.69** [a]			0.004	
Lachnoclostridium	C	2.08	1.66	0.82	0.36	**3.37**	1.000		1.000
	Br	2.50	1.68	0.53	0.31	**2.46**			
	Sy	2.16	1.59	0.88	0.29	**2.80**			
	**Mean (Age)**	**2.25** [a]	**1.64** [ab]	**0.74** [bc]	**0.32** [c]			0.001	
Faecalibacterium	C	0.72	4.51	11.41	5.93	**1.23**	0.910		0.950
	Br	1.53	6.37	13.58	6.79	**1.26**			
	Sy	3.29	4.64	12.47	5.93	**1.23**			
	**Mean (Age)**	**1.85** [bc]	**5.17** [b]	**12.49** [a]	**6.22** [bc]			0.001	
Ruminiclostridium 5	C	1.92	1.76	0.93	0.77	**5.64**	0.930		0.550
	Br	1.16	1.93	0.91	0.91	**7.07**			
	Sy	1.80	1.70	0.80	0.67	**6.58**			
	**Mean (Age)**	**1.63** [a]	**1.80** [a]	**0.88** [b]	**0.78** [b]			0.001	
Anaerotruncus	C	2.12	0.46	0.24	0.08	**1.35**	0.910		0.250
	Br	1.00	0.70	0.11	0.10	**1.23**			
	Sy	1.76	0.64	0.17	0.09	**1.24**			
	**Mean (Age)**	**1.63** [a]	**0.60** [b]	**0.18** [b]	**0.09** [b]			0.001	
Negativibacillus	C	1.58	1.69	0.78	0.69	**0.72**	0.910		0.120
	Br	1.17	2.79	0.59	0.52	**0.48**			
	Sy	2.03	2.11	0.76	0.54	**0.66**			
	**Mean (Age)**	**1.60** [a]	**2.20** [a]	**0.71** [b]	**0.58** [b]			0.001	
Lactobacillus	C	1.67	3.03	10.95	15.45	**1.18**	0.930		0.020
	Br	0.31	0.91	10.87	21.61	**1.27**			
	Sy	1.74	1.73	8.02	20.85	**1.36**			
	**Mean (Age)**	**1.24** [b]	**1.89** [b]	**9.95** [c]	**19.30** [a]			0.001	
Bacillus	C	1.53	0.89	2.07	0.95	**7.78**	0.890		0.980
	Br	0.87	0.72	1.28	0.63	**8.43**			
	Sy	0.82	0.64	1.27	0.85	**8.09**			
	**Mean (Age)**	**1.07**	**0.75**	**1.54**	**0.81**			0.190	
Oscillibacter	C	0.73	1.66	0.27	0.28	**1.36**	0.890		0.089
	Br	0.68	1.01	0.29	0.24	**0.88**			

	Sy	1.78	1.01	0.39	0.27	**0.89**			
	**Mean (Age)**	**1.06**^{a}	**1.23**^{a}	**0.31**^{b}	**0.26**^{b}			0.001	
Ruminococcaceae UCG-005	C	1.42	0.75	3.25	3.00	**0.73**	0.990		0.220
	Br	0.42	2.65	2.27	3.33	**0.55**			
	Sy	0.74	1.65	1.65	3.93	**0.86**			
	**Mean (Age)**	**0.86**^{c}	**1.68**^{bc}	**2.39**^{ab}	**3.42**^{a}			0.001	
GCA 900066575	C	1.20	1.74	0.53	0.34	**2.11**	0.910		0.210
	Br	0.55	2.24	0.49	0.24	**2.17**			
	Sy	0.76	0.76	0.72	0.26	**1.99**			
	**Mean (Age)**	**0.84**^{ab}	**1.58**^{a}	**0.58**^{b}	**0.28**^{b}			0.001	
Erysipelatoclostridium	C	0.94	0.38	1.63	1.79	**0.95**	0.450		0.100
	Br	0.22	0.27	1.36	0.81	**0.88**			
	Sy	0.93	0.29	1.66	0.84	**0.62**			
	**Mean (Age)**	**0.70**^{c}	**0.31**^{bc}	**1.55**^{a}	**1.15**^{ac}			0.001	
DTU089	C	1.01	1.47	0.46	0.24	**1.19**	0.990		0.890
	Br	0.31	2.16	0.64	0.27	**0.67**			
	Sy	0.40	1.76	0.34	0.27	**0.93**			
	**Mean (Age)**	**0.57**^{b}	**1.80**^{a}	**0.48**^{b}	**0.26**^{bc}			0.002	
Blautia	C	0.41	0.21	1.37	2.08	**0.79**	0.920		0.920
	Br	0.16	0.18	1.63	1.95	**0.84**			
	Sy	0.53	0.14	1.41	2.40	**0.69**			
	**Mean (Age)**	**0.37**^{b}	**0.18**^{b}	**1.47**^{c}	**2.14**^{a}			0.001	
CHKCI001	C	0.25	0.19	3.25	2.69	**1.02**	0.910		0.870
	Br	0.09	0.15	3.98	4.46	**0.98**			
	Sy	0.04	0.06	3.62	2.52	**1.12**			
	**Mean (Age)**	**0.13**^{b}	**0.13**^{b}	**3.62**^{a}	**3.23**^{a}			0.001	
Christensenellaceae R 7 group	C	0.20	0.15	1.25	1.69	**1.60**	1.000		0.890
	Br	0.07	0.33	1.06	1.87	**2.17**			
	Sy	0.06	0.43	1.12	1.77	**1.56**			
	**Mean (Age)**	**0.11**^{b}	**0.30**^{b}	**1.14**^{c}	**1.77**^{a}			0.001	
Romboutsia	C	0.06	0.03	1.65	4.61	**0.82**	0.890		0.091
	Br	0.02	0.04	1.18	3.80	**0.83**			
	Sy	0.05	0.06	0.77	5.15	**0.84**			
	**Mean (Age)**	**0.04**^{b}	**0.04**^{b}	**1.20**^{c}	**4.52**^{a}			0.001	
Alistipes	C	0.01	8.90	10.41	5.37	**1.59**	0.990		0.830
	Br	0.06	7.10	8.52	7.33	**1.26**			
	Sy	0.00	8.62	8.15	6.61	**1.51**			
	**Mean (Age)**	**0.02**^{b}	**8.21**^{b}	**9.02**^{c}	**6.44**^{a}			0.001	
Turicibacter	C	0.00	0.00	0.22	2.89	**6.17**	0.890		0.180
	Br	0.00	0.00	0.12	2.26	**5.75**			
	Sy	0.00	0.01	0.34	3.28	**5.84**			
	**Mean (Age)**	**0.00**^{b}	**0.00**^{b}	**0.23**^{b}	**2.81**^{a}			0.001	
Akkermansia	C	0.00	0.00	2.59	0.49	**0.78**	0.990		1.000
	Br	0.00	0.01	2.78	0.77	**0.60**			
	Sy	0.00	0.04	2.89	0.82	**0.91**			
	**Mean (Age)**	**0.00**^{b}	**0.01**^{b}	**2.75**^{a}	**0.69**^{b}			0.001	
Bacteroides	C	0.00	0.71	2.61	6.44	**0.77**	0.940		0.230
	Br	0.00	0.00	6.77	3.93	**0.89**			
	Sy	0.00	0.00	9.45	3.31	**0.94**			
	**Mean (Age)**	**0.00**^{b}	**0.24**^{b}	**6.28**^{a}	**4.56**^{a}			0.001	
Ruminococcaceae UCG-008	C	0.00	0.00	0.00	2.08	**2.44**	0.910		0.900
	Br	0.00	0.02	0.00	2.38	**2.68**			
	Sy	0.00	0.00	0.00	1.78	**3.19**			
	**Mean (Age)**	**0.00**^{b}	**0.01**^{b}	**0.00**^{b}	**2.08**^{a}			0.001	
Candidatus Arthromitus	C	0.45	0.07	0.02^{A}	0.00	**0.14**	0.910		0.950
	Br	0.18	0.08	0.01^{AB}	0.00	**0.07**			
	Sy	0.23	0.05	0.00^{B}	0.00	**0.07**			
	**Mean (Age)**	**0.29**^{a}	**0.07**^{ab}	**0.01**^{ab}	**0.00**^{b}			0.062	

Averages of the columns with different capital letter superscripts and values within the mean rows with different lowercase letters are significantly different ($p < 0.05$).

Table S11. Spearman's correlation between gut microbiota composition and host IBD virus ELISA titers on day 7-21.

Phylum	Order	Family	Genus	IC	IM	CC
Actinobacteria	Corynebacteriales	Corynebacteriaceae	*Corynebacterium 1*	-0.61		
	Micrococcales	Micrococcaceae	*Rothia*	-0.69		
			Kocuria	-0.65		
	Propionibacteriales	Propionibacteriaceae	*Cutibacterium*		0.69	
Bacteroidetes	Bacteroidales	Bacteroidaceae	*Bacteroides*			-0.72
		Rikenellaceae	*Alistipes*			-0.62
Firmicutes	Bacillales	Bacillaceae	*Anaerobacillus*		0.62	
		Planococcaceae	*Lysinibacillus*		-0.76	
		Staphylococcaceae	*Staphylococcus*	-0.68		-0.72
	Lactobacillales	Carnobacteriaceae	*Trichococcus*	-0.6		
		Lactobacillaceae	*Lactobacillus*			-0.66
		Leuconostocaceae	*Leuconostoc*	-0.76	-0.61	-0.66
			Weissella			-0.61
		Streptococcaceae	*Lactococcus*	-0.74		
			Streptococcus			-0.66
	Clostridiales	Christensenellaceae	*Christensenellaceae R7 group*			-0.77
		Defluviitaleaceae	*Defluviitaleaceae UCG-011*			-0.6
		Family XIII	*Eubacterium brachy group*			-0.78
			Family XIII UCG-001			-0.77
			Family XIII AD3011 group			-0.71
			Eubacterium nodatum group			-0.67
		Lachnospiraceae	*CHKCI001*	-0.61		-0.65
			Marvinbryantia			-0.66
		Peptostreptococcaceae	*Romboutsia*			-0.76
		Ruminococcaceae	*Ruminococcaceae UCG-004*			-0.78
			Ruminococcaceae UCG-010			-0.77
			Ruminococcus 1			-0.74
			Ruminococcaceae NK4A214 group			-0.67
			Ruminiclostridium 1			-0.65
			Faecalibacterium			-0.64
			Anaerotruncus			0.64
			Papillibacter			-0.61
	Erysipelotrichales	Erysipelotrichaceae	*Turicibacter*	-0.73		-0.71
Proteobacteria	Sphingomonadales	Sphingomonadaceae	*Sphingomonas*	0.62		
	Betaproteobacteriales	Burkholderiaceae	*Aquabacterium*	0.6		
	Enterobacteriales	Enterobacteriaceae	*Escherichia-Shigella*			0.67
	Pseudomonadales	Moraxellaceae	*Acinetobacter*	0.76		
Verrucomicrobia	Verrucomicrobiales	Akkermansiaceae	*Akkermansia*	-0.65		-0.72

Table shows only strong R > 0.6 correlation coefficients based on Spearman's rank correlation (Spearman's rho).

Table S12. Spearman's correlation between gut microbiota composition and host Gumboro titer/ parameters on day 21-40.

Phylum	Order	Family	Genus	IC	IM	CC
Actinobacteria	Bifidobacteriales	Bifidobacteriaceae	*Bifidobacterium*	0.79	0.85	0.62
			Neoscardovia		0.68	
	Micrococcales	Micrococcaceae	*Rothia*	-0.65	0.6	
	Coriobacteriales	Eggerthellaceae	*CHKCI002*			-0.75
Firmicutes	Bacillales	Planococcaceae	*Lysinibacillus*		-0.8	
		Staphylococcaceae	*Staphylococcus*	-0.61		
	Lactobacillales	Aerococcaceae	*Globicatella*	0.6		
		Carnobacteriaceae	*Carnobacterium*		-0.6	
		Lactobacillaceae	*Lactobacillus*		0.75	0.71
		Leuconostocaceae	*Leuconostoc*	-0.64		
		Streptococcaceae	*Lactococcus*	-0.68		
	Clostridiales	Clostridiaceae 1	*Candidatus Arthromitus*		-0.74	-0.67
			Clostridium sensu stricto 1	-0.83		0.73
			Clostridium sensu stricto 10	-0.66		
			Clostridium sensu stricto 12	-0.76		
		Family XI	*Gallicola*	0.68	0.72	
		Lachnospiraceae	*CHKCI001*	-0.64		
			Eubacterium fissicatena group	-0.66		
			Eubacterium hallii group	-0.62		
			Lachnoclostridium	-0.61		
			Ruminococcus torques group	-0.63		
		Peptostreptococcaceae	*Romboutsia*	0.61	0.62	0.68
		Ruminococcaceae	*Butyricicoccus*			-0.68
			Faecalibacterium			-0.61
			Hydrogenoanaerobacterium			-0.78
			Oscillospira			0.81
			Ruminococcaceae UCG-008		0.73	0.77
			Ruminococcaceae UCG-010			0.59
			Ruminococcaceae UCG-014	-0.78		
			Ruminococcus 2			0.72
	Erysipelotrichales	Erysipelotrichaceae	*Turicibacter*	0.72	0.67	0.77
Proteobacteria	Rhizobiales	Rhizobiaceae	*Ochrobactrum*		-0.8	
	Rhodospirillales	uncultured	*Azospirillum sp. 47 25*			0.76
	Desulfovibrionales	Desulfovibrionaceae	*Bilophila*			0.78
	Betaproteobacteriales	Burkholderiaceae	*Delftia*		-0.72	
			Parasutterella			0.85
	Enterobacteriales	Enterobacteriaceae	*Serratia*		-0.81	
	Pseudomonadales	Pseudomonadaceae	*Pseudomonas*		-0.72	
	Xanthomonadales	Xanthomonadaceae	*Stenotrophomonas*		-0.71	
Verrucomicrobia	Verrucomicrobiales	Akkermansiaceae	*Akkermansia*	-0.75		

Table shows only strong R > 0.6 correlation coefficients based on Spearman's rank correlation (Spearman's rho).
